# Physical activity interventions for older adults – an overview of systematic reviews

**DOI:** 10.1186/s12889-025-25002-2

**Published:** 2026-01-06

**Authors:** Jo Canneaux, Richard A. Sharpe, Noreen Orr, Jane R. Smith, Alison Bethel, Cassandra Phoenix, Victoria A. Goodwin, Iain Lang, Ruth Garside

**Affiliations:** 1 European Centre for the Environment & Human Health, Department of Public Health and Sports Science, University of Exeter Medical School, Peter Lanyon Building, Penryn Campus, Penryn, Cornwall TR10 9FE UK; 2 Faculty of Health and Life Sciences, University of Exeter Medical School, Exeter, UK; 3 Department of Sport and Exercise Sciences, Durham University, Durham, UK; 4 National Institute for Health and Care Research (NIHR) Applied Research Collaboration South West Peninsula, Exeter, UK; 5 Public Health, Cornwall Council, Truro, Cornwall UK; 6 South West Public Health Training Programme, Bristol, UK

**Keywords:** Older people, Physical activity intervention, Overview of systematic reviews

## Abstract

**Background:**

The proportion of people meeting recommended physical activity (PA) guidelines declines with age. Older adults who are physically inactive have an increased risk of: all-cause mortality; chronic diseases; injury and reduced cognitive functioning. Multiple systematic reviews aim to understand the effectiveness of PA interventions but the evidence is fragmented and it is unclear how well the research reflects the needs of older people. We conducted an overview of existing systematic reviews (PROSPERO reference: CRD42015023796) to map evidence on interventions to encourage older people to be more active.

**Methods:**

Nine electronic databases were searched, most recently in October 2023. Older people were defined as those aged 50+, with physical activity including active daily living (walking the dog, gardening, etc.), organised activities and clubs and more formal exercise or sport. Screening of records, data extraction, and assessments of quality (using AMSTAR-2) and inequality (using Progress-Plus) were completed independently by two reviewers.

**Results:**

A total of 35 reviews (reported in 36 papers) published between 2002 and 2023 were included. Reviews included between 3 and 79 studies (total 480 unique studies) with a median of 162 and 184 participants per study in meta-analyses (overall AMSTAR-2 median quality low) and narrative syntheses (median quality critically low) respectively. Eighteen included a mixture of interventions (e.g. supporting lifestyle change, walking groups, exercise classes), nine focussed on remote delivery, six on wearable devices, five on 1-to-1 interventions, two on walking, and two focussed on group-based interventions. Interventions to increase physical activity in older people were shown to be effective in the short term (< 12 months). The use of wearable devices as interventions had a small-to-medium effect on increasing physical activity and daily steps. Remote delivery approaches, including the use of text messaging, web- or mobile-technology, and social media, were effective in increasing PA.

**Conclusions:**

A range of interventions are effective in increasing PA in older adults. Newer research highlights the usefulness of technology - including wearable devices, social media, text messaging - as useful tools. Improved effectiveness seems to relate to theory-based interventions, but without further research, caution should be taken when interpreting the effectiveness of individual behaviour change techniques. Identifying and resolving wider barriers and facilitators, social and environmental interactions influencing PA may improve future interventions. Better reporting of equity by future studies will improve understanding of who benefits from such interventions. Increased knowledge of potential wider social, economic and environmental determinants of physical activity in older adults, and specifically more vulnerable and minority populations, will help policy makers and practitioners develop more effective interventions.

**Supplementary Information:**

The online version contains supplementary material available at 10.1186/s12889-025-25002-2.

## Background

Being physically active includes a range of activities from sports and structured exercise to informal movement such as walking to household tasks such as gardening or hoovering [1]. Older adults who are physically inactive have an increased risk of all-cause mortality, an increased risk of developing chronic diseases (including cardiovascular disease, cancer and diabetes), an increased risk of injury (including fractures and falls) and reduced cognitive functioning (including Alzheimer’s disease) [2]. The greatest health gains are seen in increasing weekly physical activity from 0 to 100 min [3].

Older adults are one of the fastest growing population groups in western societies [4]. Consequently, supporting older people to meet minimum guidelines and for physical activity (PA) has the potential to alleviate a substantial proportion of the societal and economic burden of major non-communicable diseases worldwide [5]. For example, even low levels of moderate-to-vigorous intensity physical activity (MVPA) can reduce mortality by 22% in older people, with those changing from doing no MVPA to doing some (1–499 Metabolic Equivalent of Task (MET)-minutes per week) seeing the greatest risk reduction [6]. 

Despite overwhelming evidence supporting the benefits of older people being physically active, the proportion meeting sufficient levels of PA globally is decreasing [7] and declines steeply in older people across all global regions, with women being less likely to be physically active than men [7–9]. Women may have lower PA than men due to environmental and personal factors which are associated more with women such as fear of falling or being violated, financial concerns, or living alone [10, 11]. Global efforts to address PA levels have led to World Health Organisation (WHO) targets to reduce the global prevalence of physical inactivity by 15% from 2018 levels by 2030 [12] These also specify an action to improve the provision of PA for older adults [12]. 

Multiple systematic reviews exist aiming to understand the effectiveness of PA interventions in older adults [9, 13–18], however, the evidence is fragmented on what exactly works for this age group, how well the research reflects the needs of older people, and how policy makers can support an increase in PA. An overview of systematic reviews is a logical step to synthesise current evidence and map what is known about the effectiveness of PA interventions in older adults [19]. This allows us to identify gaps/limitations in the literature to inform future research and provide evidence-based information to support policy and practice.

Existing overviews of reviews focussing on similar PA in older adults have not fully explored all PA interventions and what works in community living healthy older adults. They have been limited by geography [20] or age [15], to those with chronic diseases [14], or by specific intervention type (mHealth/eHealth interventions [17], behavioural change techniques [18], the built environment [9]. Consequently, this overview is unique in its approach to understanding what interventions increase PA in healthy adults aged >50 years and removing these previous limitations and instead taking a comprehensive approach in considering any interventions which could increase PA globally in healthy older adults.

## Aims

We identified and mapped existing systematic reviews on interventions aimed at increasing older people’s PA. Specifically, we aimed to address:

1. What is known about the effectiveness of interventions to encourage older people to be physically active?
2. Are there groups of older people for whom these interventions work better or less well?
3. What intervention types and features are associated with effectiveness?
4. What gaps and limitations are there in the existing evidence base?

## Methods

This overview of systematic reviews was conducted in accordance with our published protocol (PROSPERO reference: CRD42015023796; work stream 2) and reported in accordance with PRISMA 2020 guidelines (Supplementary Material 1). Working with our expert panel on physical activity in older age and experts by experience group, we defined older people as those aged 50 and over. This age group was chosen in response to input from our public engagement group, to capture the range of life experiences adults experience as they transition into older age which have the potential to impact on willingness and ability to be physically active. There were two amendments to the protocol: studies of all ages which have sub-group analyses of older adults were excluded due to the lack of high-quality data reported; and update searches were streamlined (see below and Supplementary Material 2).

### Searching and screening

Search strategies were developed by an information specialist (AB) in collaboration with the wider research team (JC, RAS, NO, CP & RG) and with input from an Expert Advisory and Experts by Experience Groups. Database searches were carried out in 2015, 2016, 2018 and then updated in 2023. The databases were chosen by the team, expert panel, experts by experience group and prior systematic reviews in this field of interest. Nine databases were searched: Amed via EBSCOhost, Cochrane: CDSR, Cochrane: DARE; Embase via Ovid; Epistemonikos; Medline via Ovid; PubMed; Sportsdiscus via EBSCOhost, Web of Science (SCI, SSCI, SHCI, CPCI-S and CPCI-SSH.

The search strategy used a combination of free text terms and relevant controlled vocabulary (e.g. MeSH) terms. The databases searched changed through the years due to new databases becoming available, databases not being updated and the search summary table results, see Supplementary Material 2. The search strategy was amended for the 2023 search using the evidence from the previous iterations. Forward citation searching was carried out in 2023.

Details of the decisions made and the search strategy changes are in Supplementary Material 2.

Screening was managed in Endnote version X7.0 [2015, 2016, 2018] (Thomas Reuters, New York, NY) and in Rayyan (Ouzzani, 2016) and recorded using the PRISMA guidelines [21]. The screening of titles and abstracts, and of full texts, was performed in duplicate with two reviewers from a pool of seven researchers (JC, RAS, RG, AB, NO, CP, JS) undertaking this independently. Disagreements were discussed and discrepancies resolved with the working group. A list of excluded full-text studies, with reasons, is included in Supplementary Material 3.

#### Inclusion criteria:

- Population: Adults aged 50 + years (or with a mean age across studies of 50+) from the general population who are living in the community.
- Interventions: Any interventions, in any settings, aimed at increasing levels of general PA or exercise.
- Comparators: Included studies with any comparison group or comparison to no intervention.
- Outcomes: Changes in PA or exercise.
- Study design: Systematic reviews of effectiveness studies (defined as reviews with explicit, repeatable approaches to identifying, including, appraising and synthesising information on primary studies).

#### Exclusion criteria:

- Population: Adults aged < 50+ (or with a mean age across studies of < 50+); reviews focussed on PA for people with specific conditions or risk factors (e.g. Rehabilitation after a cardiac event, those at risk of falls or fall prevention such as those focusing on flexibility, strength training and balance).
- Interventions: Interventions not aiming to increase PA.
- Outcomes: Reviews which included interventions targeting multiple behaviours (such as PA and dietary change) where the impact of PA could not be extracted separately.
- Reviews not published in English.
- Study designs other than systematic reviews of effectiveness.

### Data extraction

A standardised data extraction template, designed for the study, was used to capture review characteristics (inclusion criteria, number and type of studies included, sample demographics etc.); review processes (number of databases searched, assessment of bias/study quality, analysis techniques; type and nature of interventions included (e.g. delivery format, duration and intensity, content, use of theory), and relevant findings including from any relevant sub-group analyses. Data was extracted in duplicate by one reviewer (RAS, JC) and checked by another (NO, JS, RAS).

### Quality appraisal & consideration of equity issues.

The methodological quality of the included reviews was independently assessed in duplicate by three reviewers (JC, RAS & NO) using the AMSTAR-2 checklist [22] (Supplementary Material 4) which categorised the reviews as of high, moderate, low, or critically low quality. The AMSTAR-2 tool consists of 16 questions, with seven being critical domains in assessing quality. The tool was adapted for this overview by removing one of the seven critical domains: *“Did the review authors provide a list of excluded studies and justify the exclusions?”* as it was not felt to be a critical indicator of methodological quality in this overview (only one included systematic review [23] reported this). The remaining six critical domains were assessed in line with the AMSTAR-2 template (Supplementary Material 4), which included assessing the risk of bias for included primary studies.

The PROGRESS-Plus framework was used to examine whether studies reported research findings and sample characteristics by social groups that may impact on health inequality (Supplementary Material 8). This describes 13 core categories, including place of residence, deprivation, ethnicity, employment/education, family support, disability and other characteristics of vulnerable populations [24]. The PROGRESS-Plus assessment was completed independently and in duplicate by two reviewers (JC, RS, NO) using a template (Supplementary Material 4). Any disagreement between the reviewers in final AMSTAR-2 or PROGRESS-Plus scores were resolved by discussion with two additional reviewers (RG & CP) (Supplementary Material 5).

### Analysis

As this is an overview of systematic reviews, we adopted a mapping methodology to identify what has been studied and where new research is needed [25]. To consolidate the characteristics and findings of included systematic reviews, we extracted data into standardised tables. We then synthesised this evidence to provide a summary of PA intervention effectiveness (Table 1), the participants (Table 2), characteristics of interventions (Table 3), and intervention behaviour change techniques (Table 4). To maintain consistency in reporting Standard Mean Differences (SMD) from included reviews conducting meta-analyses, we interpreted SMDs as indicating small (≥ 0.20), medium (≥ 0.50), or large effects (≥ 0.80) [26]. Presenting the findings along with their quality rating enabled us to assess how much weight we applied to the findings of included systematic reviews. Reporting of heterogeneity and potential risk of bias was taken into consideration of the AMSTAR-2 assessments described above. Due to our narrative synthesises, it was not appropriate to undertake sensitivity analyses.

Reviews were grouped according to their method of synthesising results, meta-analyses and narrative syntheses, to emphasise those reviews that provided the most relevant, detailed and robust evidence of effect. They were then ranked according to their quality, as indicated by their AMSTAR-2 score. Consideration was also given to the design and quality of the studies included in these reviews when considering the robustness of findings.

As this review of systematic reviews synthesises information across a large topic area, it is likely to include overlap of primary studies and over-representation of some primary studies could lead to double counting and over-stating some outcomes. There is no standard methodological approach to account for primary study overlap [27], so a matrix was created to detail all primary studies included in the reviews (Supplementary Material 6) and a ‘corrected covered area’ (CCA) [28] was used. The CCA was calculated as: (N - r)/((r x c) - r), where ‘r’ is the number of unique citations (representing the rows of the matrix), ‘c’ is the number of reviews (columns), and N is the total number of citations in the area (r x c). CCA thresholds were categorised as: 0% = none; <5% = slight; <10% = moderate; <15% = high; >15% = very high overlap. Overlap of studies has been considered with review quality in each sub-section and where substantial duplication was identified, reviews were omitted for those subsections using the following considerations [1] the amount of overlap and whether excluding the review would omit any themes not discussed in included reviews [2], the AMSTAR-2 rating and removing reviews with large overlap where they were of the lowest quality, [3] the date the review was published where quality ratings were equal and excluding the oldest review. Any reviews which have been omitted in sub-sections due to overlap are discussed.

### Expert advisory group

The overview of systematic reviews was conceived and developed by experts in evidence synthesis. The first phase development of this overview of systematic reviews was conducted with active involvement from an Expert Advisory Group (EAG), including 14 members of the public (experts by experience group), commissioners and practitioners. EAG supported the development of the grant application and design of the overview of systematic reviews. The project team then met with EAG on a quarterly basis throughout the implementation of the study. Progress and initial findings were presented and discussed at each meeting, which included how best to disseminate the study outputs. This formed part of the co-design, which was essential and helped to guide its implementation from concept to completing the study. We also provided monthly reports to keep members up to date and to aid discussions at quarterly meetings.

## Results

**Fig. 1:**
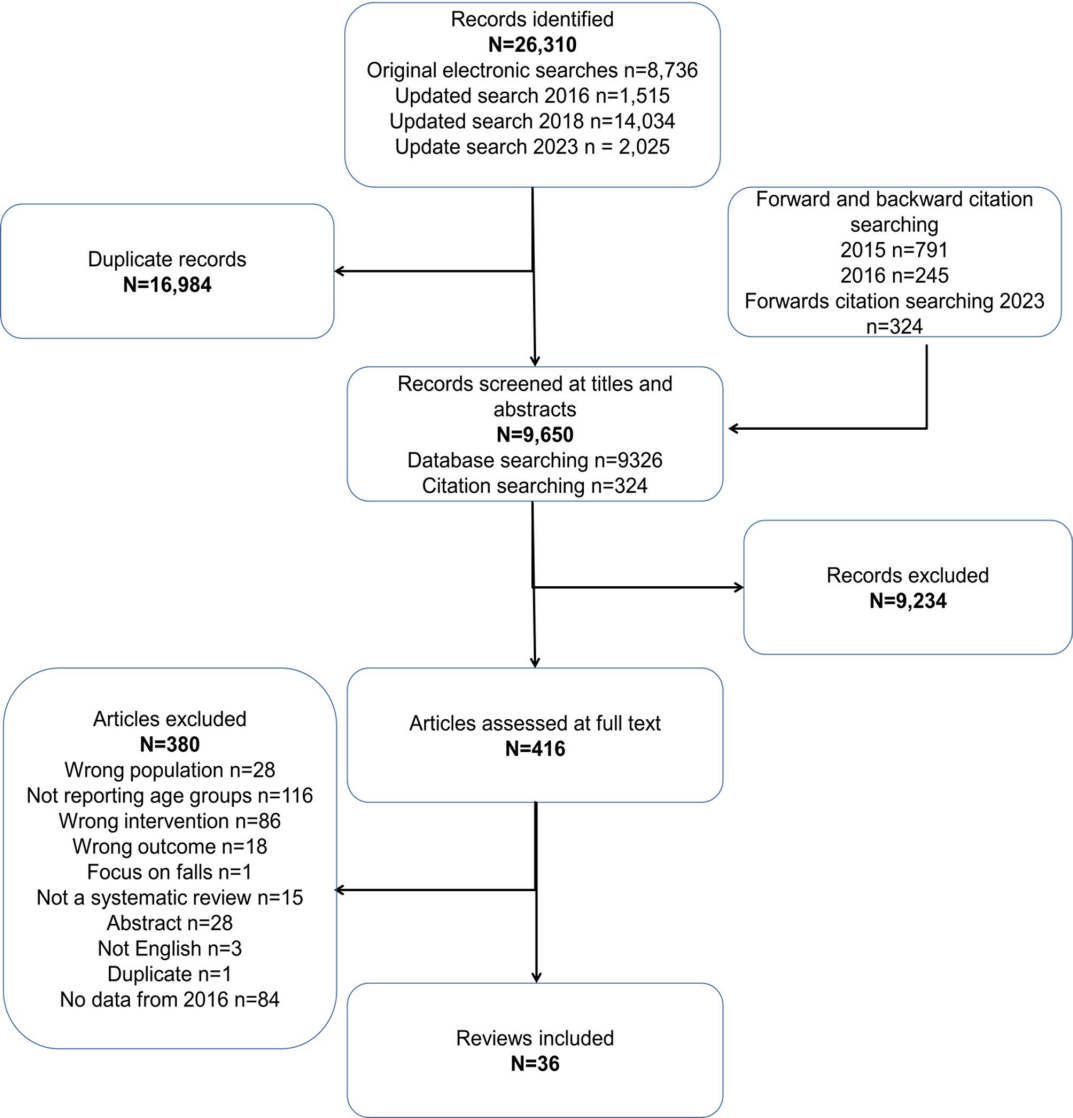
PRISMA flow diagram

Searches identified 26,310 articles (Fig. 1). We obtained 416 full texts following screening of titles and abstracts, of which 35 reviews reported in 36 articles met the inclusion criteria. One meta-analysis focussed on older adults [29] reported a second meta-analysis confined to a sub-set of the included trials [30]. A list of excluded full-text studies and reason for exclusion is included in Supplementary Material 3.

### Characteristics of included systematic reviews

As shown in Figs. 1 and 36 articles (reporting on 35 reviews) focused on studies of older adults aged 50 + years 18 of which reported meta-analyses [23, 29–42] and 17 of which conducted narrative syntheses only [20, 33, 43–57]. Reviews were published between 2002 and 2023, with search periods ranging from 1960 to 2022 and searches covering between one [33] and 12 [46] databases (five to six on average).

Reviews included a total of 480 unique studies, ranging from three [55] to 79 [31] per review and with a median number of participants per included study of 184 (39 to 770) in meta-analyses, and 162 (31 to 1372) in narrative syntheses (Table 2). The CCA of all primary studies included was 0.14%, indicating slight overlap. A total of 73.8% of studies were only included once across reviews (*n* = 355). There were some primary studies which appeared multiple times across the reviews: Wijsman et al. [58] appeared in seven reviews; King et al. [59] appeared in eight reviews, and Kolt et al. [60] appeared in nine reviews. These primary studies focus on web- and telephone-based interventions and were included in systematic reviews focussing on any type of intervention, and those focussing more specifically on web- and/or telephone-based interventions.

A total of 28 reviews reported on the country of origin of included studies, with the majority (50.4%), stemming from North America (USA and Canada), 29.8% from European countries (including The Netherlands, UK, Belgium and others), 12.2% from Oceania, 6.9% from Asia (including Japan, Taiwan and others), 0.6% from South America (Chile and Brazil), and 0.2% from South Africa (Supplementary Material 7).

Quality and reporting of equity issues (AMSTAR-2 and Progress-Plus).

Overall, the median quality of the meta-analyses and narrative syntheses were rated Low and Critically Low respectively (Supplementary Material 4). No included reviews were rated as High quality. Five meta-analyses [29, 30, 35, 38, 41, 61]and two narrative syntheses [48, 50] were rated as moderate quality. Seven meta-analyses [23, 31, 33, 39, 40, 62, 63] and five narrative syntheses [43, 50, 52, 55, 56] were rated as low. The remaining six meta-analyses [32, 34, 36, 37, 42, 64] and ten narrative syntheses [20, 33, 44–47, 49, 53, 54, 57] were rated as critically low. The most common critical weaknesses across all reviews were a lack of information about: a priori protocols (18/35, question 2); accounting for risk of bias in the interpretation and discussion of the results (18/35 question 13); and accounting for publication bias (9/18 meta-analyses question 15).

Equity issues, examined using PROGRESS-Plus checklist, were also poorly reported. No review provided any information about participants’ religious background, or sexual orientation and only one review reported on each of: area deprivation, employment status, income-related measures, community-family support, emotional/mental disability, or other vulnerable populations. Small numbers reported on education attainment levels (*n* = 4), socioeconomic status (*n* = 4), physical disability (*n* = 3). More reviews reported on age (*n* = 34), gender (*n* = 27), and ethnicity (*n* = 12) (Supplementary Material 5).

### Characteristics of populations (Table 1)

**Table 1:** Summary of characteristics of participants in studies included (AMSTAR2 rating: M = moderate; L = Low; CL = Critically Low)

	Meta-analyses (N=19 unless indicated)	Narrative syntheses (N=17 unless indicated)	Sub -group analyses
**Number of participants per included study**			
Median across reviews	184	162 (n=15)	**N/A**
Range of medians across reviews	39 – 770	31-1372	
**Age (years) of participants in included studies**	**N=18 reported any info**	**N=16 reported any info**	
Median across reviews	68.5 (N=9)	64 (N=6)	M: NS diff by age for PA with wearable activity trackers [Larsen, Wu] M: Significantly lower MVPA wearable activity trackers in older participants [Wu] M: Significant direct effect on increasing PA in 2/3 interventions which used age-stereotype based interventions [Knight] L: NS diffs by age [Chase] CL: walking ints sig greater effectiveness in older (60+) [Kassavou] CL: NS diffs by age [Conn, 2002]
Range of medians across reviews	59.8 - 75.8 (9)	59.6 - 68 (N=6)	
**Gender of participants in included studies**	**N=18 reported any info**	**N=11 reported any info**	
Median % women across reviews	63% (N=12)	67% (N=7)	M: No significant effect of gender affecting PA outcomes for wearable devices [Larsen] L: ints similarly effective regardless of % female [Chase] CL: walking ints sig. less effective in female only samples [Kassavou]
Range of medians % women across reviews	51.37-76% (N=12)	54-78.9 (N=7)	
**Ethnicity of participants in included studies**	**N=5 reported any info**	**N=8 reported any info**	
Median % white across reviews	71 % (4) (range 61 –85%)	Range 0 – 79% (3)	L: Both structured and unstructured interventions saw increased PA levels [Pedersen] CL: Limited evidence of increased PA in Asian American older adults by selecting culturally appropriate delivery modes and behaviour change techniques [Katigbak]
Number of reviews specifically aimed at non-white populations	0	2 (Asian Americans; American Indian and Alaska Native)	
Number of reviews including studies specifically aimed at non-white populations	4/19 (1)	1/64(1) 9/9 (1), 1/7 (1), 3/3 (1)	
**SES**	**N=1 reported any info**	**N=5 reported any info**	
Number of reviews including studies with low SES participants	1	2	No studies discussed the impacts of SES status on PA intervention levels
**Clinical characteristics of participants in included studies**	**N=17 reported any info**	**N=13 reported**	
Included studies in healthy samples only	N=3	N=5	M: NS diff between clinical and healthy samples [Oliveira 17, Wu, Oliveira 20] L: ints sig more effective in healthy vs. chronic illness samples [Chase] CL: NS diff between clinical and healthy samples [Oliveira 20] CL: ints sig more effective in patient vs. non-patient samples [Conn, 2002]
Included studies in samples with risk factors for chronic conditions	N=3	N=2	
Included studies in samples with chronic conditions	N=11	N=6	

The reviews primarily included studies in which participants were in their 50 s and 60 s, with only four meta-analyses [31, 33, 38, 64] and four narrative syntheses [44, 46, 49, 50] restricting inclusion to studies of participants aged, or with a mean age, of 65 years or older. Where this could be ascertained, the median age of participants across studies in the meta-analyses was 68.5 years (median range 59.8 [36] to 75.8 [64] and 64 years (median range 59.6 [20] to 68 [57]) in narrative syntheses.

The majority of reviews (30/35) explicitly provided at least some information about the health status of participants in included studies. Three meta-analysis [23, 34, 35] and five narrative syntheses [43, 51, 53, 56, 65] included only studies in healthy community-dwelling adults. Two meta-analysis [29–31] and two narrative syntheses [43, 52] also included studies in adults with risk factors for chronic disease (e.g. overweight [31], sedentary behaviou [43, 52]. A further 11 meta-analyses and six narrative syntheses additionally included a number of studies (2/19 [36] to 14/23 [42]) in older people with pre-existing conditions, including participants with diabetes [20, 37, 42, 47, 54, 61, 65] and those living in nursing homes [33, 49, 57]. 

The majority (29/35) of reviews in older adults provided information on participant gender, with a median percentage of women in meta-analyses of 63% (*n* = 12, range 51% [23] to 76% [34]) and in narrative reviews of 67% (*n* = 7, range 54% [56] to 78% [48]). Eight reviews included between one [33, 50] and twelve [57] women-only studies. Only four reviews reported inclusion of studies with all male cohorts with between 1 and 3 primary studies included [40, 41, 43, 57]. 

Thirteen reviews (five meta-analyses) reported any information on ethnicity, with the majority of participants reported as White. Five reviews included one to four studies targeting specific ethnic minority groups [32, 36, 43, 50, 51]. Two reviews specifically targeted ethnic minority groups of American Indian and Alaska Natives [55], and Asian Americans [47]. Only six narrative syntheses in older adults gave any detail on any socio-economic characteristics, and this was limited [33, 40, 43, 51–53] (Supplementary Materials 5 and 8).

### Characteristics of PA interventions and comparators (Table 2)

**Table 2:** Summary of evidence on effectiveness of different types of PA interventions in older people by type and quality of review

Review type & quality	Any PA intervention N=18	Walking interventions N=2	Wearable devices N=6	1-to-1 including primary-care based N=5	Remotely delivered N=9	Group-based N=2
**Meta-analyses in older adults**						
High N = 0	*Note: no meta-analyses rated high using AMSTAR2*					
Moderate N= 5	**Small effect on any** **PA (SMD 0.29, CI 0.19 to 0.40)** [O’Brien n=19 trials with true controls] *Small effect on self-reported PA at 12m (SMD 0.19, CI 0.10 to 0.28)* * [Hobbs n=11]* *Large effect on steps at 12m (SMD 1.08, CI 0.16 to 1.99)* * [Hobbs n=4]* **Small effect on PA with short term** (<3m) follow up (SMD 0.30, CI 0.17 to 0.43) and **intermediate term** (3-12m) follow up (SMD 0.27, CI 0.06 - 0.49) but **no effect with long-term** (12m+) follow up (SMD 0.19, CI -0.03 to 0.41) [Grande] **PA based interventions more effective compared with minimal intervention in increasing moderate-vigorous PA (MVPA) at intermediate follow up (**MD 8.80, CI 4.09 to 13.52),** but no significant difference at short-term **(MD 5.19, CI -0.61 to 10.99) [Grande] **PA-based interventions more effective than minimal intervention in increasing steps per day in short-term (<3m) **(MD 1567.01, CI 885.89 to 2248.13). [Grande]		**Medium effect on PA (SMD 0.54, CI 0.34 to 0.73), 1297 more steps (817 to 1753)** [Larsen n=21] **Small effect on increasing** **total daily PA (SMD 0.21, CI 0.01 to 0.40) ** [Wu] **Small effect of increased MVPA (SMD 0.34, CI 0.15 to 0.52). Increasing an average daily MVPA 5.5 minutes per day (2.4 to 8.4) ** [Larsen n=8] **Medium effect on increasing** **weekly MVPA (SMD 0.54, CI 0.36 to 0.72)** [Wu] **Medium effect on increasing** **daily steps (SMD 0.59, CI 0.44 to 0.75)** [Wu]	**Small effect of health coaching on PA (SMD 0.27, CI 0.18 to 0.37)** [Oliveira, 2017 N=27 trials with true controls] *Larger effect for face-to-face (SMD 0.41,CI 0.25 to 0.58, n=9) than phone-delivered (SMD 0.21, 0.11 to 0.32* *, n=18) [Oliveira, 2017]*		
Low N= 7	**Small effect on any PA (SMD 0.18, CI 0.10 to 0.26)** [Chase n=53 comparative studies] **No significant effect for workplace interventions to increase PA **Frequency of PA/exercise (per week) (SMD 0.25, CI -0.07 to 0.56) [high heterogeneity (54%) and very low quality of evidence (n=3)] No significant effect for MVPA (SMD 0.22, CI -0.05 to 0.50 [moderate heterogeneity (46.2) and low quality evidence (n=2)] [Merom]		**Small-to-medium effect on PA for accelerometers (SMD 0.43, CI 0.19 to 0.68) ** [Cooper n=4] **No significant effect on PA for pedometers (SMD 0.22, CI -0.08 to 0.51)** [Cooper n=4] **No significant effect for WAT-based interventions on MVPA (comparison with passive control) (SMD 0.22, CI -0.62 to 1.06)** [Liu n=3] **Large effect on step count for comparison with a passive control (SMD 1.23, CI 0.75 to 1.70 n=2, low quality of evidence) and for comparison with active control (SMD 1.27, CI 0.51 to 2.04, n=4, moderate quality of evidence) ** [Liu]		**Small effect for Digital Based Behavioural Change on PA in RCT studies (SMD 0.28, CI 0.01 to 0.56, n=8) and pre-post studies (SMD 0.25, CI 0.09 to 0.41, n = 6)** [Stockwell] **Small to medium effect for Digital Based Behaviour Change on MVPA (SMD 0.47, CI 0.32 to 0.62, MD = 52 min/week) ** [Stockwell] **Small to medium effect on MVPA min/day in E-health (MD 7.41, CI 3.24 to 11.57); MVPA min/week (MD 56.35, CI 17.43 to 95.27)** [Nunez de Arenas-Arroyo] **Medium effect on steps/day in ** **E-health (ES = 0.59, CI 0.15 to 1.02) Increasing an average daily steps of 1616.28 (386.25 to 2846.31)** [Nunez de Arenas-Arroyo] **No significant effect of M-Health app increases in daily step count with short term (3m) (506 steps/day, CI −80 to 1092)) or medium term (6m)** ** (753 steps/day; CI −147 to 1652) ** [Yerrakalva] *Small to medium effect of reduced sedentary time (SMD −0.45, CI −0.69 to −0.19) * *[Stockwell]*	
Critically Low N= 6	**No significant effect on any PA (SMD 0.26,CI 0.24 to 0.76)** n=43 studies, 17 trials [Conn 2002] **Small effect on any PA (SMD 0.14, CI 0.09 to 0.20)** n=16 studies any design [French] **No significant effect on PA when compared to active controls (SMD 1.55, CI -0.46 to 3.57, n=3) or non-active controls (SMD 0.18, CI -0.01 to 0.37, n=6)** [Sansano-Nadal] *Small effect on PA at six-month follow up when compared to non-active control (SMD 0.30, CI 0.15 to 0.44, n=4), but no significant effect with active controls (SMD 1.89, CI -0.89 to 4.67, n=3) * *[Sansano-Nadal]* *No effect on PA at 2 year follow up for either active controls (SMD 1.43 (CI -0.44 to 4.30) n=2) or non-active controls (SMD 0.03 (CI -0.18 to 0.24) n=2)* *[Sansano-Nadal] *	**Medium effect on any PA (SMD 0.52, CI 0.32 to 0.71)** n=19, 9 trials [Kassavou]	**Medium effect size for PA (SMD 0.55, CI 0.40 to 0.70), 1558 more steps (1099 to 2018)** [Oliveira, 2020 n=23] *No significant effect on PA at three-month follow up (SMD 0.14, CI -0.08 to 0.35)* *[Oliveira, 2020 n=4]* *Medium effect size on PA at six-month follow up (SMD 0.69, CI 0.16 to 1.23)* *[Oliveira, 2020 n=4]* *Significant difference (p=0.02) between short-term interventions <12 weeks (SMD=0.14, CI -0.26 to 0.54, n=5) and long-term interventions >12 weeks (SMD=0.70, CI 0.47 to 0.93, n=18)* *[Oliveira, 2020]*		**Significant increase in PA time when measured by PA questionnaires for E-Health 53.2 min/week (30.18 to 76.21)** [Kwan] ** Significant increase in PA time when measured by wearable devices 12.95 min/day (10.09 to 15.82), 790 more steps (300 to 1280** [Kwan]	
**Narrative syntheses in older adults**						
High N = 0	*Note: narrative syntheses rated high using AMSTAR2*					
Moderate N = 2	↑ **PA in 2/3 (66%) **in interventions which used age-stereotype based interventions [Knight]				↑ **objective PA in 5/7 (71%), subjective PA in 11/13 (85%)** trials of e-health vs. no int or other ints [Muellmann] ↑ **PA in 7/8 (88%) studies any design of ‘phone-based** [Baxter] ↑ **PA in 4/6 (67%) trials of web-based** [Muellmann] ↑ **PA in 1/2 (50%) trials of telephone-based** [Muellmann] ↑ **PA in 3/4 (75%) trials of text messaging ** [Muellmann]	
Low N= 5	↑ **PA in 4/7 (57%) **studies, 3 trials of community-based interventions [Moore] ↑ **PA in 37/54 (69%)** studies any design [Baxter] ↑ **PA in 3/3 (100%) **in American Indian and Alaska Native Older Adults [Pedersen]		↑ **PA in 4/4 (100%) **studies any design using accelerometers/pedometers [Baxter]	↑ ***PA in 7/10 (70%)*** *studies any design of individual counselling/advice [Baxter]* ↑ ***PA in 5/6 (83%)*** *studies any design of individual exercise programs [Baxter]* ↑ **PA in 2/5 (40%)** trials of tailored, primary care based [Stevens]	↑ **PA in 14/16 (88%)**, 11 trials of non face-to-face [Muller & Khoo]	↑ *PA in8/9 (89%) *studies* any design [Baxter]*
Critically Low N= 10	↑ **PA in 10/17 (59%)** trials [Conn 2003] ↑ **PA in 7/11 (64%)** trials [Van der bij] ↑ **PA in 6/8 (75%) **studies, 7 comparative [Cyarto] ↑ **PA in 6/8 (75%)** trials in nursing homes [Jansen] **Mixed evidence in 2/2 **studies that volunteer-led PA interventions increase PA levels [Lim] ↑ **PA in 'Most studies' (no specific number available) **with varying levels of cultural adaptation [Katigbak] *Limited evidence the volunteer-led PA interventions can improve other health outcomes e.g. functional status.* *[Lim]*	↑ *PA in 4/6 (67%) trials [Conn 2003]*		↑ **PA in 7/10 (70%) **studies any design of primary care based [Neidrick]	↑ **PA in 6/6 (100%)** trials of targeted messaging [Ostrander] ↑ **PA in 13/17 (76%) **pre-post interventions for mobile-technology [Elavsky] ↑ **some aspect of PA in 17/29 (59%)** RCTs [Elavsky] ↑ **PA or increased participation in 8/9 (89%) **social marketing interventions [Goethals] ↓*sedentary behaviour in 4/4 pre-post studies;* *No effect in sedentary behaviour in 5/10 (50%) RCT studies* *[Elavsky]*	↑ *PA in 4/4 (100%) trials [Van der bij]*
Bold = headline findings from reviews; *Italics* = findings from subgroup analyses in reviews. Effect size for SMD: small (*≥* 0.20); medium (*≥* 0.50); large (*≥* 0.80)						

Of the eighteen meta-analyses focussing on older adults, seven included any intervention aimed at increasing PA [29–32, 34, 35, 40, 64], five focussed on wearable devices [38, 39, 42, 61, 66] four on interventions delivered remotely [23, 37, 62, 63], one on walking interventions [36], and one on health coaching [41]. Of the seventeen narrative syntheses, ten included any PA intervention [33, 43, 44, 46–50, 55, 57], five focussed on interventions delivered remotely [20, 45, 51, 52, 54], and two focussed on primary care based interventions [53, 56]. 

The majority of meta-analyses (16/18) gave details of the comparators in the reviewed studies: seven used usual care, minimal or no intervention [23, 29, 30, 35, 38, 41], five used any active or passive control [39, 40, 42, 62, 64], three included other active interventions or pre-intervention as the comparator [36, 65, 66], and two related to e-health compared against no e-health, less advanced e-health, or a different app [37, 63]. Over half of the seventeen narrative syntheses did not report on comparators [20, 33, 44, 45, 47, 52–54, 57], six used any active or passive control [43, 46, 48, 55, 56], one compared against non e-health interventions or no intervention [51], and one compared to interventions not led by peers [49]. 

Reviews included studies targeting a range of physical activities, delivered by different intervention providers, in a range of settings and using variable delivery formats, though these were not consistently reported. The intervention duration across all reviews was extremely variable, ranging from one day to four years and with many interventions including multiple contacts lasting from 8 to 129 min. Of the reviews providing information about the design and delivery of interventions, these involved diverse delivery formats (31/35), multiple intervention providers (e.g. health professionals, exercise trainers, self-delivered) (18/35) across multiple settings including home-based (*n* = 12), community- based (*n* = 13), health or exercise facility based (*n* = 11), nursing home based (*n* = 1), and virtual (*n* = 1) interventions. (See Table 3 for details).

The most commonly reported behavioural change theories used to inform interventions were Social Cognitive Theory (13/20 reviews reporting on use of theory) and the Transtheoretical model (14/20). Reviews reported the use of behaviour change techniques, most commonly goal setting (22/35), self-monitoring (18/35), feedback (14/35), barrier management (8/35), problem solving (7/35) and using rewards/incentives (5/35). The majority of reviews (19/35) reported the use of some form of tailoring (e.g. individual goal setting, feedback and exercise prescriptions) or the individualisation of messages in included interventions (e.g. information about opportunities in the local environment) (Table 3).

**Table 3:** Characteristics of interventions in studies included focussed on physical activity interventions in older adults and their associations with effectiveness in reported sub-group analyses (AMSTAR2 rating: M = moderate; L = Low; CL = Critically Low)

	Meta-analyses (N=19 unless indicated)	Narrative syntheses (N=17 unless indicated)	Findings from meta-analyses with subgroup analyses (N=5, min. n=4 studies in any subgroup)*
**Target behaviour**	**N=16 reported**	**N=12 reported**	
Multiple health behaviours Specific type of activity	35-38% (N=6) 26-63% + 1 100% walking (N=10)	14-62% (N=6) 29-63% walking (N= 5)	M:Small significant effect of health coaching [Oliveira 17] L: NS diffs [Chase] CL: PA only sig more effective [Conn, 2002]
**Providers**	**N=9 reported**	**N=9 reported**	
	Mostly health professionals, exercise specialists, peers	Mostly health professionals, 2 self-delivered only	CL/L NS diffs by provider [Kassavou, Chase] CL: Mixed evidence on volunteer-led PA interventions in increasing PA levels [Lim]
**Setting**	**N=14 reported**	**N=11 reported**	
Home-based component Community-based component	17-78% (N=6)	17-37% (N=4 +2 home only)	M/L: diffs by setting [Hobbs, Chase] L: No definitive evidence of effectiveness of workplace interventions but on-site supervised sessions during the workday had higher retention rates and good compliance [Merrom] L: Community based interventions 4/7 showed changes in PA patterns, but high risk of bias [Moore] CL: 7/10 significant improvements in primary care PA [Neidrick] CL: centre-based sig more effective than home-based [Conn, 2002]
	5-100% (N=8)	22-38% (N=4 +1 comm only)	
Health/exercise facility component	11-65% (N=7)	13-50% (N=2, +2 1⁰ care only)	
Nursing home basedVirtual	- 100% (N=1)	17% (N=1 +1 NH only)-	
**Delivery format**		**N=12 usable info**	
Face-to-face component	61-95% (N=7)	8-100% (N= +2 remote only)	M: print -ve, phone NS [Hobbs] L: audiovisual & mailed materials +ve, ‘phone NS [Chase] L: narrative: telephone 7/8 +ve [Baxter] CL: mailed materials, phone NS [Conn, 2002] M: narrative: larger effect for face to face vs telephone [Oliveira 17] L: Group delivery 8/9 +ve [Baxter] CL: Group delivery +ve [Conn, 2002] CL NS 4/4 Group delivery +ve [Van der bij] M: Significant improvements in both subjective and objective measures of e-health interventions, web-based, telephone-based, text-messaging based (Muellmann) L: Increase in step count, MVPA and PA in e-health interventions [Nunez de Arenas-Arroyo] L: not statistically significant increase in step count in e-health interventions (short and long term) [Yerrakalva] CL: Successful programmes for social media reported a success factor as the use of social marketing techniques through the implementation of a number of criteria and better understanding of core marketing concepts. [Goethal] CL: medium effect size for mobile health increasing PA in studies with available data, but 12/29 studies failed to demonstrate PA improvement. (Elavsky) CL: Successful programmes for social media included the funding of activities to remove/reduce financial barriers & building of social ties between participants in the programmes. [Goethal]
Group-based component	14-64% (N=6 +1 group only)	17-71% (N=3)	
Used written materials	9-57% (N=4)	7-100% (N=5)	
Used telephone	7-74% (N=8)	8-66% (N=7)	
Used technology in delivery (email, web-based, video, app)	5-68% (N=6 +2 e-health only +4 physical activity monitors only)	3-65% (N=5 +1 social media only)	
Used SMS/text	7-11% (N=2)	69% (N=1)	
**Duration & intensity**	**N=15 some detail**	**N=13 some detail**	
	Large variability in reporting; Total intervention duration (N=13, 1 day to 4y) session duration (N=2, 8-129 mins) & frequency (N=1) of contacts	Large variability in reporting; total duration & contact time; no., duration, frequency of contacts	M: PA based more effective than minimal intervention in +steps per day & MRPA & PA at immediate follow up, but no difference in medium or long term (Grande) M/L: NS diffs by intensity [Hobbs, Chase] CL: more intense contact +ve [Conn, 2002] M: no significant effect on outcomes by intervention length in weeks (4-52) [Larsen] M: Short term interventions had a significantly larger pooled effect than long term interventions on PA and MVPA [Wu] L: No statistical difference in interventions of <3m or >3m, although high heterogeneity and small numbers of studies included [Yerrakalva] CL: significant difference between short-term (<12 week) and long-term (>12 week) with long-term interventions having increased PA [Oliveira 2020]
**Theory/approach used**	**N= 12 reported**	**N=8 reported**	
	29-100% used Social cognitive theory & Transtheoretical model commonest	14-100% used Social cognitive theory & Transtheoretical model commonest	M: Significant direct effect on increasing PA in 2/3 interventions which used age-stereotype based interventions [Knight] L: +ve [Chase] CL: NS [Conn, 2002] CL: Strategies to enhance long-term sustainability based on social cognitive theory showed small benefits on PA levels which declined six months after the intervention cessation [Sansano-Nadal] CL: the majority of mobile interventions with some evidence of effectiveness contained theory-guided intervention development [Elavsky]
**Tailoring**	**N=7**	**N=11 **	
	4 -100% (1 digital only tailoring)	reported some in 5-100% studies	L: +ve individual exercise programs (5/6) [Baxter] CL: Cultural tailoring of PA [Katigbak]

### Reported outcomes of PA interventions

The majority of meta-analyses (15/18) included studies using self-reported as well as objective measures of PA, with four only using objective measures such as pedometers and accelerometers [35, 38, 39, 63]. The majority of narrative syntheses (12/17) combined objective and self-reported outcomes with two using objective measures only [45, 54], one using primarily self-reported measures [53], and two [33, 52] not clearly stating what outcomes were considered.

### Effectiveness of PA interventions and comparators

#### Any PA intervention

Two meta-analyses rated as Moderate quality [29, 30, 35] assessing any PA intervention found small positive effects on PA (SMD 0.29, CI 0.19 to 0.40) [30], which were estimated by combining different measurements of PA including pedometers, accelerometer, self-report of minutes of PA or energy expenditure (as defined by Hobbs et al. [29]). Grande et al. [35]. assessed PA using pooled effects of MVPA and steps/week, which were sustained in the short (SMD 0.30, CI 0.17 to 0.43) [35] and medium term (SMD 0.27, CI 0.06–0.49) [35]. There was no overlap in primary studies between Grande et al. [35] and O’Brien et al [30]. /Hobbs et al. [29]. Evidence at 12 month follow up ranged from a small non-significant effect (SMD 0.19, CI −0.03 to 0.41) [35] to a large effect on steps (SMD 1.08, CI 0.16 to 1.99) [29]. Grande et al. [35]. reported that PA based interventions, such as exercise classes or walking groups, were more effective than minimal interventions, such as advice or self-care guidance, in increasing rates MVPA per day at intermediate follow up (MD 8.80, CI 4.09 to 13.52), with small non-significant differences in the short term for MVPA (MD 5.19, CI −0.61 to 10.99) and increased steps per day (MD 1567.01, CI 885.89 to 2248.13).

Two meta-analyses rated as Low quality [31, 40] showed a small positive effect on levels of any PA (SMD 0.18, CI 0.10 to 0.26) [31] and a small non-significant effect in workplace interventions (SMD 0.25, CI − 0.07 to 0.56), with no overlap in primary studies.

The three meta-analyses rated as Critically Low quality [32, 34, 64] showed no significant effects on PA (0.26, CI 0.24 to 0.76) [32] and when compared to active controls (SMD 1.55, CI −0.46 to 3.57) [64] or non-active controls (SMD 0.18, CI −0.01 to 0.37) [64]. There was no overlap in primary studies within these reviews [34, 64, 65]. When considering intervention follow up, Sansano-Nadal et a [64] reported a small, significant effect on PA at six-months compared with non-active controls (SMD 0.30, CI 0.15 to 0.44), but no significant effect with active controls (SMD 1.89, CI −0.89 to 4.67), and no significant effect at 2 years compared to either active (SMD 1.43, CI −0.44 to 4.30) or non-active controls (SMD 0.03, CI −0.18 to 0.24).

There were nine narrative syntheses assessing any PA interventions rated as Moderate quality [48] Low quality [43, 50, 55], and Critically Low quality [33, 44, 46, 47, 57] with the majority of all included studies reporting increases in PA. There was a slight overlap in primary studies within these reviews (4.7%).

#### Walking interventions

One meta-analysis, rated critically low quality [36] focussed on walking interventions. This found a medium effect on increasing measures of PA (SMD 0.52, CI 0.32 to 0.71). One narrative syntheses, rated Critically Low quality [44], reported an increase in PA levels in 4 out of 6 studies. There was slight overlap between these two reviews (1/35 primary studies).

#### Wearable devices

Two meta-analyses focussing on wearable devices were rated Moderate quality [38, 61] and showed a small (SMD 0.21, CI 0.01 to 0.40) [61] to medium (SMD 0.54, CI 0.34 to 0.73) [38] effect on PA. Wearables had a medium effect on increasing daily steps in the review by Wu et al. [61] (SMD 0.59, CI 0.44 to 0.75), with Larsen et al. [38] reporting an additional 1297 steps per day (CI 817 to 1753). There was also a small (SMD 0.34, CI 0.15 to 0.52) [38] to medium (SMD 0.54, CI 0.36 to 0.72) [61] effect on increasing MVPA. There was high overlap between primary studies between Larsen et al. [38] and Wu et al. [61] (14.0%; 8/57).

The two meta-analyses rated as Low quality [39, 66] reported a range of outcomes, with very high overlap between their small number of primary studies (18.8%, 3/16). Cooper et al. [66] reported no significant effect on PA assessed via pedometers (SMD 0.22, CI −0.08 to 0.51), but a small-to-medium effect for those using accelerometers (SMD 0.43, CI 0.19 to 0.68). Liu et al. [39] reported no significant effect of wearable activity trackers on MVPA (SMD 0.22, CI −0.62 to 1.06), but a large effect on step count (SMD 1.23, CI 0.75 to 1.70), however it was noted that the evidence quality of studies within this review was low to moderate. One meta-analysis [42] was omitted from this sub-analysis section due to its extremely high overlap with other reviews (20/23 primary studies) and its Critically Low quality.

One narrative synthesis, rated Low quality [43], reported a sub-group analysis of interventions using wearable devices with all primary studies showing increases in PA (*n* = 4).

#### 1-to-1interventions including primary-care based

One meta-analysis focussed on 1-to-1 interventions [41] and reported a small effect of health coaching on participants’ PA (SMD 0.27, CI 0.18 to 0.37). Sub-group analyses showed a larger, statistically significant (*p* = 0.047) effect for face-to-face (SMD 0.41,CI 0.25 to 0.58) compared to telephone-delivered interventions (SMD 0.21,CI 0.11 to 0.32) [41].

One, low quality, narrative synthesis focussing on 1-to-1 interventions [56] reported an increase in PA in two of five included trials which tailored primary care-based interventions. Sub-group analysis by Baxter et al. [43] reported increases in PA in most studies following individual counselling/advice (7/10) and individual exercise programmes (5/6). One narrative synthesis, rated Critically Low quality [53], reported an increase in PA in seven out of 10 included primary care-based interventions.

#### Remotely delivered interventions

There were three meta-analyses focussed on remotely delivered interventions, all rated as of Moderate quality [23, 62, 63]. All showed increases in PA outcomes after the interventions. Stockwell et al. [62] reported small effects on PA in both trials (SMD 0.28, CI 0.01 to 0.56) and pre-post studies (SMD 0.25, CI 0.09 to 0.41), with a small-to-medium effect on MVPA (SMD 0.47, CI 0.32 to 0.62). Nunez de Arenas-Arroyo et al. [23] reported an increase in MVPA min/week of 56.35 (CI 17.43 to 95.27) and a medium effect on daily step counts (ES = 0.59, CI 0.15 to 1.02) with a WWMD 1616 steps per day (CICI 386.25 to 2846.31). Yerrakalva et al. [63] considered the short-(3 month) and medium-(6 month) term effects of remotely delivered interventions on step count and found no statistically significant improvements (506 steps/day, CI − 80 to 1092; 753 steps/day; CI − 147 to 1652). There was high overlap in included studies: Stockwell et al. [62], Nunez de Arenas-Arroyo et al. [23] and Yerrakalva et al. [63] had a CCA of 11.8% with the greatest overlap from Yerrakalva et al. [63] with 4 out of 6 studies overlapping the other two reviews, however, Yerrakalva et al. [63] was not omitted from this overview as it was the only study which provided detail on short- and medium-term effects on PA.

One critically low quality meta-analysis focussed on remotely delivered interventions [37] reported significant increases in PA assessed via subjective (SMD 53.2 min/week; CI 30.18 to 76.21) and objective measures (SMD 2.95 min/day; CI 10.09 to 15.82), SMD 790 steps; CI 300 to 1280).

One moderate quality narrative synthesis focussed on e-health delivered interventions [51] and reported increases in PA using both objective (5/7) and subjective (11/13,) measures. An increase was seen across most studies examining e-platforms which were web-based (4/6), mobile phone-based (7/8) and text messaging based (3/4) but there was mixed evidence (1/2) from telephone-based trials [51]. 

One narrative synthesis rated Low quality [52] reported increases in PA in 14 of 16 interventions. Three narrative syntheses, all rated of Critically Low quality [20, 45, 54], reported increases in PA. This included increases in trials using targeted messaging (6/6) [54], mobile-technology (14/17) [20], and social marketing interventions (8/9) [45]. A sub-group analysis by Elavsky et al. [20] reported mixed evidence on sedentary behaviour with pre-post studies reporting a reduction (4/4), but only half of RCTs showing effects (5/10). There was moderate overlap in narrative syntheses focussing on e-health interventions (7.4%, 6/81 shared studies).

#### Group-based interventions

Two narrative syntheses, one rated Low quality^1^ and one rated Critically Low quality [57], reported sub-group analyses in which group-based interventions positively impacted on PA (8/9 and 4/4 studies), with moderate overlap between the overall reviews (5.6%, 4/71 studies).

### Sub-group syntheses examining PA in different groups of older people

#### Age

Five meta-analyses [31, 32, 36, 38, 61] and one narrative synthesis [48] reported results from sub-group analyses or discussed impacts considering the age of participants in included studies (Table 2). Two reviews (one Low, one Critically Low quality) reported no significant difference in outcomes between older and younger age groups receiving any interventions to increase PA [31, 65] Kassavou et al. [36] reported that the type of intervention with greater effectiveness in older age groups (>60 year-olds) was walking. However, the quality of the studies underpinning this result was critically low.

There was moderate quality evidence of an increase in PA in 2 of 3 studies which used stereotype-based interventions to challenge self-perceptions and views on ageing [48]. Reviews examining the impact of age on wearable activity trackers were of moderate quality and reported there to be no significant difference PA outcomes [38, 61], however, in one review wearable activity trackers appeared more effective in increasing MVPA in < 70 year olds than >70 year olds [61]. 

#### Gender

Three meta-analyses [31, 36, 38] reported results from sub-group analyses by gender, with the highest quality evidence (medium and low) reporting that gender had no significant impact on PA levels in any interventions [31] or for wearable devices specifically [38]. There was low quality evidence that walking interventions were less effective in female only samples [36]. 

#### Ethnicity

Two reviews [47, 55] specifically focussed on non-white populations. Pedersen et al.’s [55] low quality review reported that both structured and unstructured interventions increased PA levels in American Indian and Alska Native older adults. Katigback et al.’s [47] critically low-quality review reported limited evidence of increased levels of PA when culturally appropriate delivery modes and behaviour change techniques were selected for Asian American populations. The five reviews indicating they included studies which specifically targeted ethnic minority groups [36, 43, 50, 51, 65] did not report any relevant sub-group analyses.

#### Socio-economic status

No studies discussed the impacts of socioeconomic status on effectiveness of PA interventions.

### Sub-group syntheses on different PA intervention features

#### Theory-based approaches and behaviour change techniques

Three reviews specifically considered theory-based interventions. The highest quality review [31] reported larger effects on PA for theory-based interventions than interventions without a stated theoretical base (*p* < 0.01). The remaining three reviews were of critically low quality. Elavsky et al. [20] reported a positive association between theory-guided intervention development and PA levels in mobile interventions in 10/13 studies. Conn et al. [65] reported no significant effect. French et al. [34] reported an overall small effect (SMD 0.14, CI 0.09 to 0.20, *p* < 0.001) on interventions including behaviour change techniques and these are explored further below.

There was inconsistent evidence about the inclusion of specific behaviour change techniques which were considered in five reviews. The two highest quality reviews (rated moderate quality) reported that goal setting techniques, barrier identification, and self-monitoring strategies had similar effects to interventions not using behaviour change techniques [30], but providing feedback^26^ and health coaching [41] had small, significant effects on increasing PA. Four reviews of low and critically low quality agreed that, compared to interventions not using them, interventions using goal setting techniques did not increase PA outcomes [30–32, 34] and three [30, 31, 34] of the four [65] reviews reported that interventions including self-monitoring did not increase PA. There was disagreement with O’Brien et al. [30] that assessing barriers and management/problem solving [31, 34] compared to interventions not using these techniques. Other techniques identified by the low and critically low quality reviews as improving PA were motivational strategies [31] and the use of rewards [31, 34]. One additional review of critically low quality reviewed whether using social cognitive theory could enhance long-term PA changes, and whilst it showed small benefits in the short term, these declined 6 months after the intervention ceased [64] (See Table 4).

**Table 4:** Intervention behaviour change techniques in studies included focussed on physical activity interventions in older adults and their associations with effectiveness in reported sub-group analyses (behaviour change techniques/strategies reported here if similarly labelled/categorised in 2 or more reviews)

Meta-analyses (N=7 unless indicated)	Narrative syntheses (N=10 unless indicated)	Findings from meta-analyses with subgroup analyses (N=4, min. n=4 studies in any subgroup)*
**N=4 reported** Average 8-9 techniques (N=2) Education/information 47-70% Self-monitoring 33-68% Barriers/prob. solving 29-63% Goal setting 35-100% (N=4) Feedback 29-58% (N=3) Social support 26-63% (N=2) Modelling 11-28% (N=2)	**N=3 usable info** Goal setting 5-100% Self-monitoring 38-57% (N=2) Feedback 38-55% (N=2) Education 13-100% (N=3) Problem solving 25-43% (N=2) Relapse prevention 19-43% (N=2)	**No. techniques: **Inconsistent evidence that associated with effectiveness. 1 M: no. overall & no. self-regulatory techniques NS [Hobbs] 1 CL: 10+ -ve [French] **Self-monitoring:** Inconsistent findings re: association with effectiveness. 1 M, 1 L: NS [Hobbs, Chase] 1 CL: +ve [Conn, 2002] 1 CL: -ve [French] **Modelling:** Some evidence that not associated with effectiveness. 1 M, 1L, 1CL: NS [Hobbs, Chase, Conn, 2002] 1 CL: +ve [French] **Education about health/personal consequences:** Some evidence that associated with reduced effectiveness. 1 M, 1 CL, 1 CL: -ve association [Hobbs, Conn, 2002, French] 1 L: NS [Chase] **Goal setting:** Evidence that not associated with effectiveness. 1 M, 1 L, 1 CL quality: NS [Hobbs, Chase, French] **Feedback:** Inconsistent findings re: association with effectiveness. 1 M: +ve [Hobbs] 1 L: NS [Chase] 1 CL: -ve [French] **Problem solving:** Some evidence that associated with increased effectiveness. 1 <: NS [Hobbs] 1 L, 1 CL: +ve [Chase, French] **Social support:** Evidence that not associated with effectiveness. 1 M, 1 CL: NS [Hobbs, Conn, 2002] 1 CL: -ve [French] **Specific recommendation/prescription:** Some evidence that associated with reduced effectiveness. 1 M, 1 CL: -ve [Hobbs, French] 1 L: NS [Chase] **Prompt practice:** Some evidence that associated with reduced effectiveness. 1 M: NS [Hobbs] 1 CL: -ve [French] **Instruction on how to perform:** Some evidence that not associated with effectiveness. 1 M, 1 CL: NS [Hobbs, French] **Goal review:** Some evidence that not associated with effectiveness. 1 M, 1 CL: NS [Hobbs, French] **Follow up prompt:** Some evidence that not associated with effectiveness. 1 M, 1 L: NS [Hobbs, Chase]

#### Providers

The type of provider was not shown to affect intervention effectiveness in two reviews of low and critically low quality [31, 36]. There was mixed evidence from a low quality reviews about whether volunteer-led interventions increased PA levels [49]. 

#### Setting

A total of six reviews considered the impact of settings on intervention effectiveness. Evidence was mixed: two reviews of moderate and low quality found no impact [30, 31], while one review [32] reported that interventions delivered at exercise centres were more effective than exercising at home, although this review was rated as of critically low quality. Two narrative reviews (low and critically-low quality) had positive results in the majority of their studies where interventions were based in the general community [50] and in primary care [53]. One low quality review [40] specifically reported on workplace interventions, and showed no significant effect on increasing PA (SMD 0.25, CI −0.07 to 0.56) or MVPA (SMD 0.22, CI −0.05 to 0.50).

#### Delivery format

There was evidence from four reviews of moderate and low quality that interventions increase PA levels when delivered via audio visual methods, mailed materials, and via telephone [29–31, 43]. One moderate quality review [41] reported larger effects on PA from face-to-face compared to telephone delivery. There were positive PA increases in low and critically low reviews in group-based interventions compared to a control [43, 57, 65]. One critically low review identified increases in PA where social ties were built between participants in the programmes, and that the use of incentives or reduction of financial barriers, e.g. free exercise interventions [45]. 

More recently published reviews of moderate and low quality reported generally positive results in relation to e-health delivery compared to no control, no intervention, non e-health intervention or a modified dose of an intervention, ranging from showing significant improvements in subjective and objective measures [51] and increased step-counts [23] to small non-statistically significant step count increases (WMD 506 steps/day; 95% CI − 80 to 1092) [63]. One critically low-quality review [20] reported a medium effect on increasing PA with mobile-health delivery, but 12/29 studies failed to demonstrate an improvement [20]. 

#### Duration & intensity

Grande et al., [35] a review of moderate quality, reported greater initial impacts on PA, MVPA, and step-count from PA interventions over more minimal interventions (usual healthcare, advice, waiting list, and self-care guidelines) but these were not sustained at medium and long-term follow up. Conn et al. [65] (critically low quality), assessed the impact of activity intensity, reporting greater impact with moderate than low intensity PA interventions.

Six reviews considered the impact of intervention duration on PA outcomes, with four reviews reporting that intervention duration did not influence PA outcomes [29, 31, 38, 63]. Wu et al. [61] a moderate quality review, reported that short-term interventions had a larger effect on PA & MVPA than longer term interventions. One critically low-quality review reported that long-term interventions had increased effects over short term [41]. 

## Discussion

### Effectiveness of interventions on encouraging physical activity

This overview examined the effectiveness of interventions promoting PA in older adults (over 50 years of age) from existing systematic reviews. Previous overviews have been narrower in scope in terms of, for example, age and intervention type, so this review is unique in terms of its broad scope. We included 36 papers reporting 35 reviews assessing the effectiveness of interventions aimed at increasing physical activity in adults aged 50 and older. These reviews included studies of over 270,000 people across 480 primary studies, representing a vast amount of investment and data collection on this topic. Overall the overlap between studies included in the reviews was slight (CCA 0.14%).

Generally, the evidence shows that interventions are effective at increasing older people’s physical activity levels in the short term (< 12 months), based on MVPA and steps-per-day. We were unable to fully investigate PA outcomes in the long-term, which have been previously found to decline [67]. Other studies have found a range of factors influencing PA, which includes elements such as community safety, social interactions, motivation/enjoyment, misperceptions of PA, affordability, socio-cultural ageing stereotypes, self-efficac and perceived health gains [68–70]. The use of technology in interventions is also effective with wearable devices having a small to medium effect on increasing PA and daily steps and delivery methods such as text messaging, web-based, mobile-technology and social marketing all increasing PA, MVPA and step counts. This is positive and has the potential to result in a range of health benefits [29]. Evidence on longer term impacts (12 months+) was limited and mixed.

Despite the amount of research and research synthesis undertaken in this area, interpreting how to precipitate such positive changes, in which contexts and among which populations is complex, with limited detailed information about the nature of population and interventions included in the reviews. The reported effect ranges may be due to a number of factors. Most reviews combined subjective and objective measures of PA [71]. Self-reported physical activity duration is subject to a range of biases and may result in a greater variation in effect sizes [72]. Step counting using pedometers was shown by one included review to result in higher levels of physical activity; [29] it is not clear whether this is because use of pedometers in themselves constitute more effective interventions, or because they are more accurate measures of change. Changing physical activity behaviour is a complex, multifaceted phenomenon [73]. The identification of interventions and components which suit different groups of people, in relation to age, gender, SES and health status, could provide an opportunity to improve future strategies. Additionally, the use of conceptual frameworks such as the 24-hour model [74] of movement could allow for a more holistic understanding of how interventions have impacted functional health through the lifespan.

### Effectiveness of intervention targeting different participant characteristics

Generally, the reporting of which populations were included in the studies being systematically reviewed was poor. Where such details were reported, it seems that white, women in their 60 s were the most commonly studied. Few of the PROGRESS-plus criteria, which outlines the population characteristics that should be considered in reviews focusing on issues of equity, were reported.

There was extremely limited evidence on the impact of ethnicity on physical activity interventions, which may be due to fewer primary studies reporting this information or targeting interventions to ethnically diverse populations, which is consistent with other current evidence [13]. 

No studies discussed the impacts of socioeconomic status on changes in physical activity levels. It is unknown whether the findings of this review can be appropriately applied to other populations or whether the results would be the same in more marginalised groups. Needs may be different in, for example, men, older people, those from isolated communities, some ethnic groups and people living in supported housing or residential care. This is further compounded by inconsistent reporting across reviews and some contrasting findings. It is also possible that these findings are due to sample size limitations and/or differences in perceived barriers and facilitators (e.g. time/capacity to perform physical activity and health status [75]) of physical activity. With advancing age, people’s perception of barriers and facilitators may change, highlighting the need to address individual needs on health benefits, participant’s fears, preferences, social support and any constraints related to the physical environment [76]. Furthering our understanding of wider social and environmental determinants of physical activity should form the basis of future research [75]. In doing so, this will help inform the tailoring of interventions (e.g. information about the environment and physical activity [29, 54],)which was shown to have a positive association with increased PA [43, 47, 56]. This needs to account for different needs of older people for example, women may have lower PH than men. Moreover, increasing perceptions of personal relevance [52] and self-efficacy [34] towards physical activity may help improve the effectiveness of interventions.

### Effectiveness of specific intervention components

Generally, the reviews give few details about the composition, delivery or timeframe of interventions that were found to be successful, but moderate intensity PA was found to have a greater impact that low intensity and minimal interventions [35, 44]. It appeared that using personalised step-count goals was better than using time-based goals, and that addressing physical environmental determinants of physical activity may increase intervention effectiveness [29]. Socio-environmental determinants to consider include both individual and group-based walking, good weather, location (e.g. enjoyable scenery) and planning. It is notable that none of the included reviews evaluated the impact of environmental or policy level changes on older people’s activity levels.

Excluding more social, cultural and environmental determinants from interventions may also help explain some of the inconsistent evidence associated with intervention features. Furthermore, we found some evidence suggesting that interventions were effective regardless of the type of provider. However, positive social interactions or experiences with providers have been previously found to be an important facilitator [77]. Whilst there is mixed evidence on where PA changes were best achieved, community-based settings, including exercise centres and primary care settings, were acceptable settings in achieving positive changes to PA [44, 50, 53]. There was no definitive evidence on workplace interventions, which is supported by wider evidence on workplace interventions [78]. 

Our findings supported the use of a range of delivery methods, such as text messaging, web-based, mobile-technology and social marketing in increasing PA, MVPA, and step counts [20, 23, 37, 45, 51, 52, 54, 62, 63]. There was evidence of wearable devices having of a small to medium effect on increasing PA and daily steps [38, 61]. E-health interventions had mixed results, but some significant improvements were reported in PA and step counts [20, 23, 51, 63]. Mixed results may be due to the acceptability of technology in older age and among different communities, which includes wider determinants of health such as digital exclusion [17]. Despite this challenge, the systematic reviews have provided some evidence of effectiveness. There may be administrative and economic benefits for such interventions compared to face-to-face and group/community-based methods, which were also found to be effective approaches [36, 43, 50, 57]. However, for many people social aspects of activity are important and there were increases to PA where social connections were built [45]. To be successful, remote delivery interventions may need to be complimented by personal contact to account for individual care needs [52]. Incorporating the views and preferences of older people has the potential to achieve more effective physical activity interventions and long-term adherence rates [50]. Other delivery factors which saw improvements in PA were free/reduced cost interventions [45] which can apply to both the costs of equipment needed for remote interventions, or the costs for attending interventions at a location.

In common with some previous work on theory-based interventions [79], we found mixed findings based on use of theory in intervention design [20, 30, 31, 34, 41, 44, 48]. This may be due to the use of some inefficient individual theory components (e.g. stages of change) [79] and/or a focus on cognitive versus behavioural strategies [44]. Effective behaviour change components were motivational strategies, use of rewards, barriers management/problem solving [31, 34] and addressing age-stereotyping [48]. There was some evidence that PA levels significantly increased when supported by health coaching [41]. Interventions incorporating other behaviour change techniques had mixed findings and require further investigation. This may be due to decreases in executive function, such as planning, in older adults meaning they may benefit less from such techniques [34]. For example, we found no evidence supporting use of behaviour change techniques such as action planning, provide instruction and reinforcing techniques, which have been found to increase physical activity in younger adults [80]. 

### Strengths and limitations in the evidence base

This review has undergone a structured approach to synthesising the highest level of evidence on the effectiveness of PA interventions [32]. It was designed and delivered with the support of an expert advisory group, which helps to ensure the research health’s needs of older adults. A number of limitations exist. The evidence is restricted by the quality of the included systematic review and of the original studies with the median quality being Low and Critically Low. Some restrictions applied across the reviews for example, the sources of funding was reported in all the systematic reviews included, however, none discussed the funding sources for any of their primary studies. This synthesis approach summarises evidence from a plethora of intervention providers, settings, type, quality and time [8], making it difficult to identify the effectiveness of specific interventions components. Differences across intervention effectiveness highlight the need for more long-term valid, accurate and reliable measures of PA [72, 81], along with better review reporting against the AMSTAR-2 and Progress-Plus criteria. Potential overlap of original interventions may introduce an element of double counting, however, efforts have been made to account for this, and overall overlap was slight. There was limited evidence on the equity of PA interventions which may be due to fewer primary studies reporting this information or the lack of interventions targeted to diverse populations. Where such details were reported it seems that white, women in their 60 s from North America were the most commonly studied. As a consequence these results may not be generalisable to some groups of people, such as different ethnicities, socio-economic backgrounds, or the oldest of old (80 + years) [31]. All interventions could be enhanced by increasing our understanding of older adults’ expectations and values of physical activity, thus promoting more positive experiences. Moreover, designing interventions to make physical activity programmes more joyful, engaging and social [82] may improve effectiveness.

### Implications for policy and practice

This review demonstrates that a range of interventions are effective in promoting PA in older adults. Newer research highlights the use of technology including wearable devices, social media, text messaging, and other electronic delivery are useful tools in increasing PA in older adults. Evidence is currently limited to short-term outcomes (< 12 months). Better understanding and reporting of equity by future studies will help to identify and resolve wider barriers/facilitators including social and environmental interactions. Increased knowledge of potential wider social, economic and environmental determinants of PA in older adults and specifically more vulnerable and minority populations will help policy makers and practitioners develop more effective interventions. These findings support the ambitions to shift from treating illness to prevention through the promotion of increased PA in older adults, which has been proposed as a biomarker for healthy ageing and reduce the burden of adverse health events, disability and mortality in older age [83]. Promoting healthy ageing is a worldwide public health priority. This overview of systematic reviews provides the latest evidence-based PA programmes for healthy living older adults. These findings will both inform policy and the national level and local practice through the development of health promotion strategies and programmes based on PA and healthy ageing interventions.

## Supplementary Information

Supplementary Material 1. PRISMA checklist

Supplementary Material 2. Search methods

Supplementary Material 3. Excluded studies

Supplementary Material 4. AMSTAR2 quality assessment

Supplementary Material 5. PROGRESS-plus

Supplementary Material 6. Included study overlap in reviews

Supplementary Material 7. Country / continent of included studies

Supplementary Material 8. Data extraction

## Data Availability

All data and materials will be published with the manuscript and made available via the journals online supplementary material.

## Abbreviations

BMI: Body Mass Index
CI 95%: Confidence interval
MVPA: Moderate-to-vigorous physical activity
PA: Physical activity
SCT: Social cognitive theory
SES: Socio-economic status
SMD: Standardised mean difference
TTM: Transtheoretical model
