# Supplementary material for: Physical activity interventions for older adults – an overview of systematic reviews
Source: BMC Public Health. 2026 Jan 6;26:205. doi: 10.1186/s12889-025-25002-2 (PMC12805782; doi:10.1186/s12889-025-25002-2)
Supplement: Supplementary file 2 — Supplementary Material 2. Search methods [file 12889_2025_25002_MOESM2_ESM.docx]

**Search methods appendix**

***Databases searched and when***

|  | 2015 | 2016 | 2018 | 2023 |
| --- | --- | --- | --- | --- |
| AMED via EBSCOhost from 1995 | X | X |  |  |
| Cochrane: CDSR from 1995 | X | X | X |  |
| Cochrane: DARE no dates found | X | X |  |  |
| Embase via Ovid from 1974 | X | X | X |  |
| Epistemonikos |  |  | X | X |
| Medline via Ovid from 1946 | X | X | X | X |
| PubMed from 1946 |  | X |  |  |
| Sportsdiscus via EBSCOhost from 1573 | X | X |  |  |
| Web of Science (SCI from1990; SSCI from 1990; AHCI from 1975; CPCI-S from 1990; CPCI-SSH from 1990) | X | X | X |  |

Forward and backward citation searching using the included articles from the database searches was undertaken in Web of Science and Scopus in February 2016

***April 2016 search:*** Date limits were used in the search and PubMed (ahead of print and not in Medline) was also searched to try and find additional relevant studies not yet in Medline.

***September 2018 search***. A search summary table was completed. AMED, Sportdiscus and PubMed were not searched as none of them provided unique studies and only retrieved one included article which was the same one and was retrieved by two of the other databases. DARE was not included in this search as it was no longer updated. A new database, Epistemonikos, which had just been launched, was included in the databases searched.

All of the included articles to date had the phrase “physical activity” either in the title or abstract, once the duplicates had been removed from the searches a simple search was done in endnote for “physical activity” in the title or abstract, the resulting hits (1,521) were double screened with the remainder (1200) single screened.

***October 2023 search.*** The SST was used again and it was discovered that all of the included articles to date were in the Medline database, although the original search was not retrieving them all. The search strategy was amended and the search was only undertaken in Medline and Epistemonikos, all the included articles to this date were retrieved by the amended search. Forwards citation searching using the included papers to date was also undertaken in Scopus, the number of hits = 2025, duplicates were then removed and a simple search in endnote was performed using the terms review or meta analysis (as all the included papers to that date used these terms), the remaining records, 324 were added to the database search results to be screened.

***Full search summary table for the project***

***Medline search strategies:***

2023

**Database: Ovid MEDLINE(R) ALL <1946 to September 27, 2023>**
**Search Strategy:**
**1**  meta analy$.tw. (279517)
**2**  metaanaly$.tw. (2617)
**3**  ((comprehensive* or systematic* or integrative) adj2 (bibliographic* or literature or review* or overview*)).tw. (383739)
**4**  exp meta-analysis/ (187158)
**5**  exp meta-analysis as topic/ (28368)
**6**  cochrane.ab. (138048)
**7**  embase.ab. (158819)
**8**  (psychlit or psyclit).ab. (917)
**9**  (psychinfo or psycinfo).ab. (60214)
**10**  (cinahl or cinhal).ab. (47433)
**11**  science citation index.ab. (3826)
**12**  reference list$.ab. (22441)
**13**  bibliograph$.ab. (22874)
**14**  hand-search$.ab. (8662)
**15**  manual search$.ab. (6189)
**16**  selection criteria.ab. (36539)
**17**  data extraction.ab. (33429)
**18**  review*.ab. (2510336)
**19**  "systematic review"/ (239943)
**20**  review*.ti. (722646)
**21**  6 or 7 or 8 or 9 or 10 or 11 or 12 or 13 or 14 or 15 or 16 or 17 (307652)
**22**  18 and 21 (208693)
**23**  1 or 2 or 3 or 4 or 5 or 19 or 20 or 22 (1002995)
**24**  Adult*.tw. (1567333)
**25**  Middle age*.tw. (63832)
**26**  older.tw,kw. (561205)
**27**  *adult/ or exp aged/ or exp middle aged/ (5573074)
**28**  aged.tw. (730433)
**29**  (physical* adj1 activ*).ti. (53906)
**30**  24 or 25 or 26 or 27 or 28 (7158565)
**31**  23 and 29 and 30 (2050)
**32**  limit 31 to yr="2018 -Current" (1207)

2015, 2016 and 2018

2015 lines 1-123.
2016 update added in Line 124 with date limits
2018 same search, lines 1-123 ie no date limit applied

Database: Ovid MEDLINE(R) In-Process & Other Non-Indexed Citations and Ovid MEDLINE(R) <1946 to Present>

Search Strategy:

--------------------------------------------------------------------------------

1 meta analy$.tw. (88264)
2 metaanaly$.tw. (1585)
3 ((comprehensive* or systematic* or integrative) adj2 (bibliographic* or literature or review* or overview*)).tw. (105260)
4 exp meta-analysis/ (64218)
5 exp meta-analysis as topic/ (14762)
6 cochrane.ab. (42200)
7 embase.ab. (42816)
8 (psychlit or psyclit).ab. (885)
9 (psychinfo or psycinfo).ab. (11474)
10 (cinahl or cinhal).ab. (14139)
11 science citation index.ab. (2359)
12 bids.ab. (385)
13 cancerlit.ab. (603)
14 6 or 7 or 8 or 9 or 10 or 11 or 12 or 13 (68158)
15 reference list$.ab. (12094)
16 bibliograph$.ab. (13262)
17 hand-search$.ab. (4796)
18 relevant journals.ab. (879)
19 manual search$.ab. (2928)
20 15 or 16 or 17 or 18 or 19 (30424)
21 selection criteria.ab. (22949)
22 data extraction.ab. (12261)
23 21 or 22 (33382)
24 Review Literature as Topic/ (6166)
25 exp "Review"/ (2099813)
26 23 and 25 (22312)
27 1 or 2 or 3 or 4 or 5 or 24 (185889)
28 Comment/ (658664)
29 Letter/ (910429)
30 Editorial/ (398865)
31 animal/ (5830714)
32 human/ (15840342)
33 31 not (31 and 32) (4191698)
34 28 or 29 or 30 or 33 (5604798)
35 14 or 20 or 26 or 27 (216468)
36 35 not 34 (204885)
37 Exercis*.tw. (223194)
38 Sport*.tw. (49438)
39 Walk*.tw. (82710)
40 Bicycle*.tw. (11167)
41 Jog*.tw. (1775)
42 Swim*.tw. (29603)
43 Danc*.tw. (4970)
44 Run.tw. (64416)
45 Running.tw. (44623)
46 yoga.tw. (2653)
47 tai chi.tw. (1070)
48 garden*.tw. (8372)
49 Pilates.tw. (224)
50 Allotment*.tw. (469)
51 Active travel*.tw. (181)
52 Cycling.tw. (41902)
53 football*.tw. (5880)
54 aerobics.tw. (475)
55 gym$1.tw. (835)
56 or/37-55 (505221)
57 physical.tw. (480456)
58 aerobic*.tw. (65500)
59 muscle*.tw. (584283)
60 flexibilit*.tw. (49736)
61 resistance.tw. (524912)
62 57 or 58 or 59 or 60 (1142282)
63 57 or 58 or 59 or 60 or 61 (1626453)
64 activ*.tw. (3785063)
65 fit*.tw. (234541)
66 educat*.tw. (426110)
67 train*.tw. (374297)
68 strength*.tw. (280545)
69 endur*.tw. (33511)
70 64 or 65 or 66 or 68 or 69 (4567647)
71 64 or 65 or 66 or 67 or 68 or 69 (4800970)
72 ((physical or aerobic* or muscle* or flexibilit*) adj2 (activ* or fit* or educat* or strength* or endur*)).tw. (126164)
73 ((physical or aerobic* or muscle* or flexibilit* or resistance) adj2 (activ* or fit* or educat* or train* or strength* or endur*)).tw. (142288)
74 56 or 72 or 73 (596977)
75 exp Sports/ (142969)
76 exercise/ or exp circuit-based exercise/ or exp cool-down exercise/ or exp muscle stretching exercises/ or exp physical conditioning, human/ or exp plyometric exercise/ or exp resistance training/ or exp running/ or exp swimming/ or exp walking/ or exp warm-up exercise/ (134610)
77 exp Physical Fitness/ (23534)
78 exp Dancing/ (2116)
79 exp Gardening/ (658)
80 74 or 75 or 76 or 77 or 78 or 79 (657012)
81 Physical*.ti. (96836)
82 fit*.ti. (26357)
83 Exercis*.ti. (93310)
84 Sport*.ti. (15776)
85 Walk*.ti. (18634)
86 Bicycle*.ti. (2178)
87 Jog*.ti. (498)
88 Swim*.ti. (8821)
89 Danc*.ti. (2751)
90 Run.ti. (3537)
91 Running.ti. (7961)
92 garden*.ti. (2476)
93 activ*.ti. (929723)
94 cycling.ti. (7806)
95 allotment*.ti. (64)
96 football*.ti. (3325)
97 aerobics.ti. (111)
98 gym$1.ti. (166)
99 yoga.ti. (1682)
100 tai chi.ti. (742)
101 flexibility.ti. (5738)
102 strengthening.ti. (2948)
103 81 or 82 or 83 or 84 or 85 or 86 or 87 or 88 or 89 or 90 or 91 or 92 or 93 or 94 or 95 or 96 or 97 or 98 or 99 or 100 or 101 or 102 (1180626)
104 Adult*.tw. (920003)
105 Men$1.tw. (405403)
106 Women$1.tw. (756725)
107 man.tw. (252558)
108 woman.tw. (173853)
109 Geriatric*.tw. (35667)
110 Senior*.tw. (28809)
111 Middle age*.tw. (37693)
112 aged.tw. (416736)
113 (Person* or people* or population*).tw. (1889241)
114 older.tw. (302778)
115 ((Person* or people* or population*) adj2 older).tw. (33918)
116 (50s or fifties or 60s or sixties or 70s or seventies or 80s or eighties).tw. (15150)
117 midlife*.tw. (3529)
118 (year* or age* or over*).tw. (6212775)
119 ((50s or fifties or 60s or sixties or 70s or seventies or 80s or eighties) adj2 (year* or age* or over*)).tw. (492)
120 elder*.tw. (200749)
121 *adult/ or exp aged/ or exp middle aged/ (4173943)
122 104 or 105 or 106 or 107 or 108 or 109 or 110 or 111 or 112 or 113 or 114 or 115 or 116 or 117 or 118 or 119 or 120 or 121 (9981158)
123 36 and 80 and 103 and 122 (3374)
124 limit 123 to ed=20150601-20160530 (428)
