# Supplementary material for: Physical activity interventions for older adults – an overview of systematic reviews
Source: BMC Public Health. 2026 Jan 6;26:205. doi: 10.1186/s12889-025-25002-2 (PMC12805782; doi:10.1186/s12889-025-25002-2)
Supplement: Supplementary file 3 — Supplementary Material 3. Excluded studies [file 12889_2025_25002_MOESM3_ESM.docx]

**Excluded articles from full text screening, original search from 2015, second updated search in 2018 and final search in 2023. The data from the 2016 update search has been lost**

|  | **Reason: Abstract only** |
| --- | --- |
| 1 | Targeted health promotion activities encourage people to walk and cycle instead of using cars. Evidence-Based Healthcare and Public Health. 2005;9(2):139-40. |
| 2 | Pedometer use increases physical activity and may promote weight loss. Journal of the National Medical Association. 2008;100(3):348-9. |
| 3 | Abariga S, Wang C. Tai chi and health related quality of life: A systematic review and meta-analysis of randomized controlled trials. BMC Complementary and Alternative Medicine. 2012;12. |
| 4 | Abi Saleh R, Lirette S, Elisson J, Wright M, Cleinman A. Nutrition, or nutrition and exercise? A systematic review of interventions in frail elderly people. J Am Geriatr Soc. 2015;63:S61. |
| 5 | Aguiar E, Morgan P, Collins C, Plotnikoff R, Callister R. The effectiveness of multi-component Type 2 Diabetes prevention programs including diet, aerobic exercise and resistance training: A systematic review and meta-analyses. Obesity Research and Clinical Practice. 2012;6:79. |
| 6 | Allen P, Auslander W. EXERCISE INTERVENTIONS TO IMPROVE BALANCE AMONG COMMUNITY-DWELLING OLDER ADULTS: A SYSTEMATIC REVIEW. Gerontologist. 2010;50:263-4. |
| 7 | Baker G, Bird E, Powell J, Mutrie N. Examining theory and evidence to inform the development of an active commuting intervention: An iConnect case study. Journal of Science and Medicine in Sport. 2012;15:S3. |
| 8 | Baker PRA, Francis DP, Soares J, Weightman AL, Foster C. Community wide interventions for increasing physical activity. Sao Paulo Medical Journal. 2011;129(6):436-7. |
| 9 | Bize R, Johnson JA, Plotnikoff R. The association between health-related quality of life and physical activity level among the general adult population: a systematic review. European Journal of Public Health. 2006;16:197-8. |
| 10 | Bize R, Johnson JA, Plotnikoff RC. The association between physical activity level and health-related quality of life in the general adult population: A systematic review. Journal of General Internal Medicine. 2007;22:110-1. |
| 11 | Chase JD, Phillips LJ, Ruppar T, Conn VS. META-ANALYSIS OF FITNESS OUTCOMES OF SUPERVISED EXERCISE INTERVENTIONS AMONG HEALTHY OLDER ADULTS. Gerontologist. 2013;53:328-. |
| 12 | Cupples M, Cleland C, Kee F, Tully M. Implementing physical activity interventions: A systematic review. European Journal of Cardiovascular Prevention and Rehabilitation. 2011;1):S10. |
| 13 | Davies C, Spence J, Vandelanotte C, Caperchione C, Mummery K. How Effective are Web-Based Programs in Increasing Physical Activity? Research Update. 2012;19(4):1-2. |
| 14 | Falk Mueller-Riemenschneider F, Nocon M, Nitzschke K, Willich SN. Point-of-choice prompts: A public health approach to increase physical activity. European Journal of Cardiovascular Prevention and Rehabilitation. 2011;1):S16. |
| 15 | Geraedts H, Zijlstra A, Stevens M, Zijlstra W. REMOTE FEEDBACK IN HOMEBASED PHYSICAL ACTIVITY INTERVENTIONS FOR OLDER ADULTS: A SYSTEMATIC REVIEW. Journal of Aging and Physical Activity. 2012;20:S81-S. |
| 16 | Haanstra TM, Kamper SJ. Increasing physical activity in healthy adults: a meta-analysis. Br J Sports Med. 2012;46(14):998-9. |
| 17 | Hindin S, Zelinski E. TRAINING FOR TRANSFER: A META-ANALYSIS OF COGNITIVE AND PHYSICAL FITNESS TRAINING IN OLDER ADULTS. Gerontologist. 2009;49:147-. |
| 18 | Kelly M, Loughrey D, Lawlor B, Robertson I, Walsh C, Brennan S. Exercise interventions for healthy older adults: A systematic review. Irish Journal of Medical Science. 2014;1):S377. |
| 19 | Klima D, Kuhn E, Fiackos K, Ethier D, Patel R, Treuth M. DANCE AND OLDER ADULTS: A SYSTEMATIC REVIEW. Gerontologist. 2010;50:337-8. |
| 20 | Lin X, Zhang X, Guo J, Roberts CK, McKenzie S, Wu WC, et al. Effects of exercise training on cardiorespiratory fitness and biomarkers of cardio-metabolic health: A systematic review and meta-analysis of randomized controlled trials. Circulation. 2015;131. |
| 21 | Llobera J, March S, Torres E, Ramos M, Ripoll J, Medina D, et al. Health-promoting community activities in primary health care. A systematic review. European Journal of General Practice. 2014;20 (3):222-3. |
| 22 | Orrow G, Kinmonth AL, Sanderson S, Sutton S. Republished research: effectiveness of physical activity promotion based in primary care: systematic review and meta-analysis of randomised controlled trials.[Reprint of BMJ. 2012;344:e1389; PMID: 22451477]. Br J Sports Med. 2013;47(1):27. |
| 23 | Patel NK, Newstead AH, Ferrer RL, Parchman ML, Lawrence VA. Effects of yoga in older adults-a systematic review. J Am Geriatr Soc. 2011;59:S172. |
| 24 | Reynolds RC, McKenzie S, Allender S, Brown K, Foulkes C. Systematic review of incidental physical activity community interventions: Results and contribution to government strategy. Obesity Research and Clinical Practice. 2014;8:86-7. |
| 25 | Rosenbaum S, Sherrington C. Is exercise effective in promoting mental well-being in older age? A systematic review. Br J Sports Med. 2011;45(13):1079-80. |
| 26 | Thompson DL. Interventions to promote walking: Systematic review. Southern Medical Journal. 2008;101(2):114-5. |
| 27 | Wayne PM, Walsh J, Taylor-Piliae R, Wells R, Papp K, Donovan N, et al. The impact of Tai-Chi on cognitive performance in older adults: A systematic review and meta-analysis. Journal of Alternative and Complementary Medicine. 2014;20 (5):A10-A1. |
| 28 | Yang G, Wang L, Ren J, Zhang Y, Liu J. Clinical research evidence of Tai Chi: A systematic literature review. Journal of Alternative and Complementary Medicine. 2014;20 (5):A62. |
|  |  |
|  |  |
|  | **Reason: Duplicate** |
| 1 | Burton E, Farrier K, Hill KD, Codde J, Airey P, Hill A-M. Effectiveness of peers in delivering programs or motivating older people to increase their participation in physical activity: Systematic review and meta-analysis. Journal of sports sciences. 2018;36(6):666-78. |
| 2 | Lim SER, Cox NJ, Roberts HC, Lim SER, Roberts HC, Cox NJ, et al. The effectiveness of volunteer-led physical activity interventions in improving health outcomes for community-dwelling older people: A systematic review. Age Ageing. 2020;49. |
|  |  |
|  |  |
|  | **Reason: Focus on falls** |
| 1 | Verhagen AP, Immink M, Meulen A, Bierma-Zeinstra SM. The efficacy of Tai Chi Chuan in older adults: a systematic review (Structured abstract). Family Practice [Internet]. 2004; 21(1):[107-13 pp.]. Available from: <http://onlinelibrary.wiley.com/o/cochrane/cldare/articles/DARE-12004006114/frame.html>. |
|  |  |
|  |  |
|  | **Reason: Wrong intervention** |
| 1 | Aarsland D, Sardahaee FS, Anderssen S, Ballard C, Alzheimer's Society Systematic Review g. Is physical activity a potential preventive factor for vascular dementia? A systematic review. Aging Ment Health. 2010;14(4):386-95. |
| 2 | Ahlskog JE, Geda YE, Graff-Radford NR, Petersen RC. Physical exercise as a preventive or disease-modifying treatment of dementia and brain aging. Mayo Clin Proc. 2011;86(9):876-84. |
| 3 | Aladro-Gonzalvo AR, Machado-Diaz M, Moncada-Jimenez J, Hernandez-Elizondo J, Araya-Vargas G. The effect of Pilates exercises on body composition: a systematic review. J Bodywork Mov Ther. 2012;16(1):109-14. |
| 4 | Anderson D, Seib C, Rasmussen L. Can physical activity prevent physical and cognitive decline in postmenopausal women? A systematic review of the literature. Maturitas. 2014;79(1):14-33. |
| 5 | Anderson LM, Quinn TA, Glanz K, Ramirez G, Kahwati LC, Johnson DB, et al. The effectiveness of worksite nutrition and physical activity interventions for controlling employee overweight and obesity: a systematic review. American Journal of Preventive Medicine. 2009;37(4):340-57. |
| 6 | Arbesman M, Mosley LJ. Systematic review of occupation- and activity-based health management and maintenance interventions for community-dwelling older adults. Am J Occup Ther. 2012;66(3):277-83. |
| 7 | Arent SM, Landers DM, Etnier JL. The effects of exercise on mood in older adults: A meta-analytic review. Journal of Aging and Physical Activity. 2000;8(4):407-30. |
| 8 | Asikainen TM, Kukkonen-Harjula K, Miilunpalo S. Exercise for health for early postmenopausal women: a systematic review of randomised controlled trials. Sports Med. 2004;34(11):753-78. |
| 9 | Baert V, Gorus E, Mets T, Geerts C, Bautmans I. Motivators and barriers for physical activity in the oldest old: a systematic review. Ageing Research Reviews. 2011;10(4):464-74. |
| 10 | Baker MK, Atlantis E, Fiatarone Singh MA. Multi-modal exercise programs for older adults (Provisional abstract). Age and Ageing [Internet]. 2007; 36(4):[375-81 pp.]. Available from: http://onlinelibrary.wiley.com/o/cochrane/cldare/articles/DARE-12007003110/frame.html. |
| 11 | Baker PR, Costello JT, Dobbins M, E BW. The benefits and challenges of conducting an overview of systematic reviews in public health: a focus on physical activity. J Public Health (Oxf). 2014;36(3):517-21. |
| 12 | Blackman KCA, Zoellner J, Berrey LM, Alexander R, Fanning J, Hill JL, et al. Assessing the Internal and External Validity of Mobile Health Physical Activity Promotion Interventions: A Systematic Literature Review Using the RE-AIM Framework. J Med Internet Res. 2013;15(10):81-95. |
| 13 | Boone-Heinonen J, Evenson KR, Taber DR, Gordon-Larsen P. Walking for prevention of cardiovascular disease in men and women: a systematic review of observational studies. Obes Rev. 2009;10(2):204-17. |
| 14 | Cancela J, Oliveira I, Rodríguez-Fuentes G. Effects of Pilates method in physical fitness on older adults. A systematic review. European Reviews of Aging & Physical Activity. 2014;11(2):81-94. |
| 15 | Carvalho A, Rea IM, Parimon T, Cusack BJ. Physical activity and cognitive function in individuals over 60 years of age: a systematic review. Clin Interv Aging. 2014;9:661-82. |
| 16 | Chang YK, Pan CY, Chen FT, Tsai CL, Huang CC. Effect of resistance-exercise training on cognitive function in healthy older adults: a review (Provisional abstract). Journal of Aging and Physical Activity [Internet]. 2012; 20(4):[497-517 pp.]. Available from: http://onlinelibrary.wiley.com/o/cochrane/cldare/articles/DARE-12012046647/frame.html. |
| 17 | Chong CS, Tsunaka M, Tsang HW, Chan EP, Cheung WM. Effects of yoga on stress management in healthy adults: A systematic review. Altern Ther Health Med. 2011;17(1):32-8. |
| 18 | Colcombe S, Kramer AF. Fitness effects on the cognitive function of older adults: a meta-analytic study. Psychol Sci. 2003;14(2):125-30. |
| 19 | Conn VS. Depressive symptom outcomes of physical activity interventions: meta-analysis findings. Ann Behav Med. 2010;39(2):128-38. |
| 20 | Drukker M, Bie RA, Rossum E. The effects of exercise training in institutionalized elderly people: a systematic review (Structured abstract). Physical Therapy Reviews [Internet]. 2001; 6(4):[273-85 pp.]. Available from: http://onlinelibrary.wiley.com/o/cochrane/cldare/articles/DARE-12005005083/frame.html. |
| 21 | Freene N, Davey R, Sathiyakumar R, McPhail SM. Can physical activity measurement alone improve objectively-measured physical activity in primary care?: A systematic review and meta-analysis. Preventive medicine reports. 2020;20:101230. |
| 22 | Frost SS, Goins RT, Hunter RH, Hooker SP, Bryant LL, Kruger J, et al. Effects of the built environment on physical activity of adults living in rural settings. Am J Health Promot. 2010;24(4):267-83. |
| 23 | Fujihira H, Kubacki K, Ronto R, Pang B, Rundle-Thiele S. Social Marketing Physical Activity Interventions Among Adults 60 Years and Older: A Systematic Review. Social Marketing Quarterly. 2015;21(4):214-29. |
| 24 | Garrett S, Elley CR, Rose SB, O'Dea D, Lawton BA, Dowell AC. Are physical activity interventions in primary care and the community cost-effective? A systematic review of the evidence. Br J Gen Pract. 2011;61(584):e125-33. |
| 25 | Geraedts H, Zijlstra A, Bulstra SK, Stevens M, Zijlstra W. Effects of remote feedback in home-based physical activity interventions for older adults: a systematic review. Patient Educ Couns. 2013;91(1):14-24. |
| 26 | Gidlow C, Johnston LH, Crone D, James D. Attendance of exercise referral schemes in the UK: A systematic review. Health Education Journal. 2005;64(2):168-86. |
| 27 | Gillison FB, Skevington SM, Sato A, Standage M, Evangelidou S. The effects of exercise interventions on quality of life in clinical and healthy populations; a meta-analysis. Soc Sci Med. 2009;68(9):1700-10. |
| 28 | Guimaraes AV, Rocha SV, Barbosa AR. Exercise and cognitive performance in older adults: A systematic review. Medicina (Brazil). 2014;47(4):377-86. |
| 29 | Haaland DA, Sabljic TF, Baribeau DA, Mukovozov IM, Hart LE. Is regular exercise a friend or foe of the aging immune system? A systematic review. Clin J Sport Med. 2008;18(6):539-48. |
| 30 | Hartley L, Dyakova M, Holmes J, Clarke A, Lee MS, Ernst E, et al. Yoga for the primary prevention of cardiovascular disease. Cochrane Database Syst Rev. 2014;5:CD010072. |
| 31 | Hartley L, Flowers N, Lee MS, Ernst E, Rees K. Tai chi for primary prevention of cardiovascular disease. Cochrane Database Syst Rev. 2014;4:CD010366. |
| 32 | Higgins TJ, Middleton KR, Winner L, Janelle CM. Physical activity interventions differentially affect exercise task and barrier self-efficacy: a meta-analysis. Health Psychol. 2014;33(8):891-903. |
| 33 | Hillsdon M, Foster C, Thorogood M. Interventions for promoting physical activity. Cochrane Database Syst Rev. 2005(1):CD003180. |
| 34 | Hindin SB, Zelinski EM. Extended practice and aerobic exercise interventions benefit untrained cognitive outcomes in older adults: a meta-analysis. J Am Geriatr Soc. 2012;60(1):136-41. |
| 35 | Hong SY, Hughes S, Prohaska T. Factors affecting exercise attendance and completion in sedentary older adults: a meta-analytic approach. Journal of Physical Activity & Health. 2008;5(3):385-97. |
| 36 | Houde SC, Melillo KD. Cardiovascular health and physical activity in older adults: an integrative review of research methodology and results. J Adv Nurs. 2002;38(3):219-34. |
| 37 | Ige-Elegbede J, Pilkington P, Gray S, Powell J. Barriers and facilitators of physical activity among adults and older adults from Black and Minority Ethnic groups in the UK: A systematic review of qualitative studies. Preventive medicine reports. 2019;15:100952. |
| 38 | Jackson NW, Howes FS, Gupta S, Doyle J, Waters E. Policy interventions implemented through sporting organisations for promoting healthy behaviour change. Cochrane Database Syst Rev. 2005(2):CD004809. |
| 39 | Kelley GA, Kelley KS, Hootman JM, Jones DL. Exercise and health-related quality of life in older community-dwelling adults: A meta-analysis of randomized controlled trials. Journal of Applied Gerontology. 2009;28(3):369-94. |
| 40 | Kelley GA, Sharpe Kelley K. Aerobic exercise and resting blood pressure in older adults: a meta-analytic review of randomized controlled trials. J Gerontol A Biol Sci Med Sci. 2001;56(5):M298-303. |
| 41 | Kelly ME, Loughrey D, Lawlor BA, Robertson IH, Walsh C, Brennan S. The impact of exercise on the cognitive functioning of healthy older adults: a systematic review and meta-analysis. Ageing Research Reviews. 2014;16:12-31. |
| 42 | Kennedy CM, Powell J, Payne TH, Ainsworth J, Boyd A, Buchan I. Active assistance technology for health-related behavior change: an interdisciplinary review. J Med Internet Res. 2012;14(3):e80. |
| 43 | Klavestrand J, Vingard E. The relationship between physical activity and health-related quality of life: a systematic review of current evidence. Scand J Med Sci Sports. 2009;19(3):300-12. |
| 44 | Knight RL, Chalabaev A, McNarry MA, Mackintosh KA, Hudson J. Do age stereotype-based interventions affect health-related outcomes in older adults? A systematic review and future directions. Br J Health Psychol. 2022;27(2):338-73. |
| 45 | Koeneman MA, Verheijden MW, Chinapaw MJ, Hopman-Rock M. Determinants of physical activity and exercise in healthy older adults: a systematic review. Int. 2011;8:142. |
| 46 | Kredlow MA, Capozzoli MC, Hearon BA, Calkins AW, Otto MW. The effects of physical activity on sleep: a meta-analytic review. J Behav Med. 2015;38(3):427-49. |
| 47 | Kruger J, Buchner DM, Prohaska TR. The prescribed amount of physical activity in randomized clinical trials in older adults. Gerontologist. 2009;49 Suppl 1:S100-7. |
| 48 | Laine J, Kuvaja-Kollner V, Pietila E, Koivuneva M, Valtonen H, Kankaanpaa E. Cost-effectiveness of population-level physical activity interventions: a systematic review. Am J Health Promot. 2014;29(2):71-80. |
| 49 | Law LL, Barnett F, Yau MK, Gray MA. Effects of combined cognitive and exercise interventions on cognition in older adults with and without cognitive impairment: a systematic review. Ageing Research Reviews. 2014;15:61-75. |
| 50 | Lewis BA, Marcus BH, Pate RR, Dunn AL. Psychosocial mediators of physical activity behavior among adults and children. American Journal of Preventive Medicine. 2002;23(2 Suppl):26-35. |
| 51 | Lim SER, Cox NJ, Roberts HC, Lim SER, Roberts HC, Cox NJ, et al. The effectiveness of volunteer-led physical activity interventions in improving health outcomes for community-dwelling older people: A systematic review. Age Ageing. 2020;49. |
| 52 | Merom D, Stanaway F, Sweeting J, Tiedemann A, Mumu S, Gebel K, et al. Effects on exercise and fitness outcomes of workplace physical activity interventions targeting older employees: A systematic review and meta-analysis. ResearchSquare. 2019. |
| 53 | Miller SM, Taylor-Piliae RE. Effects of Tai Chi on cognitive function in community-dwelling older adults: a review (Provisional abstract). Geriatric Nursing [Internet]. 2014; 35(1):[9-19 pp.]. Available from: http://onlinelibrary.wiley.com/o/cochrane/cldare/articles/DARE-12014016639/frame.html. |
| 54 | Muller-Riemenschneider F, Reinhold T, Willich SN. Cost-effectiveness of interventions promoting physical activity. Br J Sports Med. 2009;43(1):70-6. |
| 55 | Muntaner A, Vidal-Conti J, Palou P. Increasing physical activity through mobile device interventions: A systematic review. Health Inform J. 2016;22(3):451-69. |
| 56 | Murphy MH, Nevill AM, Murtagh EM, Holder RL. The effect of walking on fitness, fatness and resting blood pressure: a meta-analysis of randomised, controlled trials. Preventive Medicine. 2007;44(5):377-85. |
| 57 | Netz Y, Wu MJ, Becker BJ, Tenenbaum G. Physical activity and psychological well-being in advanced age: a meta-analysis of intervention studies. Psychol Aging. 2005;20(2):272-84. |
| 58 | Nicklett EJ, Anderson LA, Yen IH. Gardening activities and physical health among older adults: A review of the evidence. Journal of Applied Gerontology. 2016;35(6). |
| 59 | Núñez de Arenas-Arroyo S, Cavero-Redondo I, Alvarez-Bueno C, Sequí-Domínguez I, Reina-Gutiérrez S, Martínez-Vizcaíno V. Effect of eHealth to increase physical activity in healthy adults over 55 years: A systematic review and meta-analysis. Scand J Med Sci Sports. 2021;31(4):776-89. |
| 60 | Park SH, Han KS, Kang CB. Effects of exercise programs on depressive symptoms, quality of life, and self-esteem in older people: a systematic review of randomized controlled trials. Appl Nurs Res. 2014;27(4):219-26. |
| 61 | Patel NK, Newstead AH, Ferrer RL. The effects of yoga on physical functioning and health related quality of life in older adults: a systematic review and meta-analysis. J Altern Complement Med. 2012;18(10):902-17. |
| 62 | Picorelli AM, Pereira LS, Pereira DS, Felicio D, Sherrington C. Adherence to exercise programs for older people is influenced by program characteristics and personal factors: a systematic review. J Physiother. 2014;60(3):151-6. |
| 63 | Rissel C, Curac N, Greenaway M, Bauman A. Physical activity associated with public transport use--a review and modelling of potential benefits. Int J Environ Res Public Health. 2012;9(7):2454-78. |
| 64 | Rogers CE, Larkey LK, Keller C. A review of clinical trials of tai chi and qigong in older adults (Structured abstract). West J Nurs Res [Internet]. 2009; 31(2):[245-79 pp.]. Available from: http://onlinelibrary.wiley.com/o/cochrane/cldare/articles/DARE-12009105017/frame.html. |
| 65 | Rolland Y, Abellan van Kan G, Vellas B. Physical activity and Alzheimer's disease: from prevention to therapeutic perspectives. J Am Med Dir Assoc. 2008;9(6):390-405. |
| 66 | Roux L, Pratt M, Tengs TO, Yore MM, Yanagawa TL, Van Den Bos J, et al. Cost effectiveness of community-based physical activity interventions. American Journal of Preventive Medicine. 2008;35(6):578-88. |
| 67 | Saunders LE, Green JM, Petticrew MP, Steinbach R, Roberts H. What are the health benefits of active travel? A systematic review of trials and cohort studies. PLoS ONE. 2013;8(8):e69912. |
| 68 | Shihui C, Yanjie Z, Hairui C, Chunxiao L. Effects of Tai Chi Exercise on People of Middle/Senior Age with Sub-Health Condition. Asian Journal of Exercise & Sports Science. 2014;11(1):55-63. |
| 69 | Smith PJ, Blumenthal JA, Hoffman BM, Cooper H, Strauman TA, Welsh-Bohmer K, et al. Aerobic exercise and neurocognitive performance: a meta-analytic review of randomized controlled trials. Psychosom Med. 2010;72(3):239-52. |
| 70 | Snowden M, Steinman L, Mochan K, Grodstein F, Prohaska TR, Thurman DJ, et al. Effect of exercise on cognitive performance in community-dwelling older adults: review of intervention trials and recommendations for public health practice and research. J Am Geriatr Soc. 2011;59(4):704-16. |
| 71 | Song Y, Qu J, Zhang D, Zhang J. Feasibility and Effectiveness of Mobile Phones in Physical Activity Promotion for Adults 50 Years and Older: A Systematic Review. Topics in Geriatric Rehabilitation. 2018;34(3):213-22. |
| 72 | Stensel D. Primary prevention of CVD: physical activity. Clin Evid (Online). 2009. |
| 73 | Stern C, Konno R. Physical leisure activities and their role in preventing dementia: a systematic review. International Journal of Evidence-Based Healthcare. 2009;7(4):270-82. |
| 74 | Suskin N, McKelvie RS. Aerobic exercise and normotensive adults: a meta-analysis. Clin J Sport Med. 1996;6(3):213. |
| 75 | Torbeyns T, Bailey S, Bos I, Meeusen R. Active workstations to fight sedentary behaviour. Sports Med. 2014;44(9):1261-73. |
| 76 | Vagetti GC, Barbosa Filho VC, Moreira NB, Oliveira V, Mazzardo O, Campos W. Association between physical activity and quality of life in the elderly: a systematic review, 2000-2012. Rev Bras Psiquiatr. 2014;36(1):76-88. |
| 77 | Valenzuela M, Sachdev P. Can cognitive exercise prevent the onset of dementia? Systematic review of randomized clinical trials with longitudinal follow-up. Am J Geriatr Psychiatry. 2009;17(3):179-87. |
| 78 | van der Deijl M, Etman A, Kamphuis CB, van Lenthe FJ. Participation levels of physical activity programs for community-dwelling older adults: a systematic review. BMC Public Health. 2014;14:1301. |
| 79 | Vasquez BA, Betriana F, Nemenzo E, Inabangan AK, Tanioka R, Garcia L, et al. Effects of Healthcare Technologies on the Promotion of Physical Activities in Older Persons: A Systematic Review. Informatics for health & social care. 2023;48(2):196-210. |
| 80 | Wang D, Macmillan T. The benefits of gardening for older adults: a systematic review of the literature (Provisional abstract). Activities Adaptation and Aging [Internet]. 2013; 37(2):[153-81 pp.]. Available from: http://onlinelibrary.wiley.com/o/cochrane/cldare/articles/DARE-12013041184/frame.html. |
| 81 | Wayne PM, Walsh JN, Taylor-Piliae RE, Wells RE, Papp KV, Donovan NJ, et al. Effect of tai chi on cognitive performance in older adults: systematic review and meta-analysis. J Am Geriatr Soc. 2014;62(1):25-39. |
| 82 | White JL, Ransdell LB, Vener J, Flohr JA. Factors related to physical activity adherence in women: review and suggestions for future research. Women Health. 2005;41(4):123-48. |
| 83 | Windle G, Hughes D, Linck P, Russell I, Woods B. Is exercise effective in promoting mental well-being in older age? A systematic review. Aging Ment Health. 2010;14(6):652-69. |
| 84 | Young J, Angevaren M, Rusted J, Tabet N. Aerobic exercise to improve cognitive function in older people without known cognitive impairment.[Update of Cochrane Database Syst Rev. 2008;(3):CD005381; PMID: 18646126]. Cochrane Database Syst Rev. 2015;4:CD005381. |
| 85 | Zheng G, Li S, Huang M, Liu F, Tao J, Chen L. The effect of Tai Chi training on cardiorespiratory fitness in healthy adults: a systematic review and meta-analysis. PLoS ONE. 2015;10(2):e0117360. |
|  |  |
|  |  |
|  | **Reason: Language** |
| 1 | De Sousa Vieira S, Teles Tavares J, Serra AJ, Shiguemitsu Suzuki F. Role of structured exercise training programs for older subjects: A systematic review. Gazzetta Medica Italiana Archivio per le Scienze Mediche. 2014;173(1-2):57-61. |
| 2 | Morin Fraile V. [Promotion of physical activity]. Rev Enferm. 2013;36(1):8-16. |
| 3 | Rivas AR, Rodríguez-Martín B. Effectiveness of multicomponent interventions to promote physical activity in the elderly: A systematic. Gerokomos. 2020;31(3):149-57. |
|  |  |
|  |  |
|  | **Reason: No age given** |
| 1 | Abioye AI, Hajifathalian K, Danaei G. Do mass media campaigns improve physical activity? a systematic review and meta-analysis. Arch. 2013;71(1):20. |
| 2 | Amiri Farahani L, Asadi-Lari M, Mohammadi E, Parvizy S, Haghdoost AA, Taghizadeh Z. Community-based physical activity interventions among women: a systematic review. BMJ Open. 2015;5(4):e007210. |
| 3 | Angevaren M, Aufdemkampe G, Verhaar HJ, Aleman A, Vanhees L. Physical activity and enhanced fitness to improve cognitive function in older people without known cognitive impairment. Cochrane Database Syst Rev. 2008(3):CD005381. |
| 4 | Anonymous. Increasing physical activity. A report on recommendations of the Task Force on Community Preventive Services. MMWR Recomm Rep. 2001;50(RR-18):1-14. |
| 5 | Appuhamy JA, Kebreab E, Simon M, Yada R, Milligan LP, France J. Effects of diet and exercise interventions on diabetes risk factors in adults without diabetes: meta-analyses of controlled trials. Diabetol Metab Syndr. 2014;6:127. |
| 6 | Ashford S, Edmunds J, French DP. What is the best way to change self-efficacy to promote lifestyle and recreational physical activity? A systematic review with meta-analysis. Br J Health Psychol. 2010;15(Pt 2):265-88. |
| 7 | Baker PR, Francis DP, Soares J, Weightman AL, Foster C. Community wide interventions for increasing physical activity. Cochrane Database Syst Rev. 2011(4):CD008366. |
| 8 | Baker PR, Francis DP, Soares J, Weightman AL, Foster C. Community wide interventions for increasing physical activity.[Update of Cochrane Database Syst Rev. 2011;(4):CD008366; PMID: 21491409]. Cochrane Database Syst Rev. 2015;1:CD008366. |
| 9 | Barr-Anderson DJ, AuYoung M, Whitt-Glover MC, Glenn BA, Yancey AK. Integration of short bouts of physical activity into organizational routine a systematic review of the literature. American Journal of Preventive Medicine. 2011;40(1):76-93. |
| 10 | Belanger-Gravel A, Godin G, Amireault S. A meta-analytic review of the effect of implementation intentions on physical activity. Health Psychology Review. 2013;7(1):23-54. |
| 11 | Bird EL, Baker G, Mutrie N, Ogilvie D, Sahlqvist S, Powell J. Behavior change techniques used to promote walking and cycling: a systematic review. Health Psychol. 2013;32(8):829-38. |
| 12 | Bize R, Johnson JA, Plotnikoff RC. Physical activity level and health-related quality of life in the general adult population: a systematic review. Preventive Medicine. 2007;45(6):401-15. |
| 13 | Bock C, Jarczok MN, Litaker D. Community-based efforts to promote physical activity: a systematic review of interventions considering mode of delivery, study quality and population subgroups. J Sci Med Sport. 2014;17(3):276-82. |
| 14 | Bort-Roig J, Gilson ND, Puig-Ribera A, Contreras RS, Trost SG. Measuring and influencing physical activity with smartphone technology: a systematic review. Sports Med. 2014;44(5):671-86. |
| 15 | Bottorff JL, Seaton CL, Johnson ST, Caperchione CM, Oliffe JL, More K, et al. An Updated Review of Interventions that Include Promotion of Physical Activity for Adult Men. Sports Med. 2015;45(6):775-800. |
| 16 | Bravata DM, Smith-Spangler C, Sundaram V, Gienger AL, Lin N, Lewis R, et al. Using pedometers to increase physical activity and improve health: a systematic review. Jama. 2007;298(19):2296-304. |
| 17 | Brown DR, Soares J, Epping JM, Lankford TJ, Wallace JS, Hopkins D, et al. Stand-alone mass media campaigns to increase physical activity: a Community Guide updated review. American Journal of Preventive Medicine. 2012;43(5):551-61. |
| 18 | Buchholz SW, Wilbur J, Ingram D, Fogg L. Physical activity text messaging interventions in adults: a systematic review. Worldviews Evid Based Nurs. 2013;10(3):163-73. |
| 19 | Bui L, Mullan B, McCaffery K. Protection motivation theory and physical activity in the general population: a systematic literature review. Psychol Health Med. 2013;18(5):522-42. |
| 20 | Bull ER, Dombrowski SU, McCleary N, Johnston M. Are interventions for low-income groups effective in changing healthy eating, physical activity and smoking behaviours? A systematic review and meta-analysis. BMJ Open. 2014;4(11):e006046. |
| 21 | Carroll JK, Yancey AK, Spring B, Figueroa-Moseley C, Mohr DC, Mustian KM, et al. What are successful recruitment and retention strategies for underserved populations? Examining physical activity interventions in primary care and community settings. Transl Behav Med. 2011;1(2):234-51. |
| 22 | Cavill JL, Jancey JM, Howat P. Review and recommendations for online physical activity and nutrition programmes targeted at over 40s. Glob Health Promot. 2012;19(2):44-53. |
| 23 | Chase JA, Conn VS. Meta-analysis of fitness outcomes from motivational physical activity interventions. Nurs Res. 2013;62(5):294-304. |
| 24 | Christian HE, Westgarth C, Bauman A, Richards EA, Rhodes RE, Evenson KR, et al. Dog ownership and physical activity: a review of the evidence. Journal of Physical Activity & Health. 2013;10(5):750-9. |
| 25 | Cleland CL, Tully MA, Kee F, Cupples ME. The effectiveness of physical activity interventions in socio-economically disadvantaged communities: a systematic review. Preventive Medicine. 2012;54(6):371-80. |
| 26 | Cleland V, Granados A, Crawford D, Winzenberg T, Ball K. Effectiveness of interventions to promote physical activity among socioeconomically disadvantaged women: a systematic review and meta-analysis. Obes Rev. 2013;14(3):197-212. |
| 27 | Coble JD, Rhodes RE. Physical activity and Native Americans: a review. American Journal of Preventive Medicine. 2006;31(1):36-46. |
| 28 | Conn VS. Anxiety outcomes after physical activity interventions: meta-analysis findings. Nurs Res. 2010;59(3):224-31. |
| 29 | Conn VS, Hafdahl AR, Cooper PS, Brown LM, Lusk SL. Meta-analysis of workplace physical activity interventions. American Journal of Preventive Medicine. 2009;37(4):330-9. |
| 30 | Conn VS, Hafdahl AR, Mehr DR. Interventions to increase physical activity among healthy adults: meta-analysis of outcomes. Am J Public Health. 2011;101(4):751-8. |
| 31 | Conn VS, Phillips LJ, Ruppar TM, Chase JA. Physical activity interventions with healthy minority adults: meta-analysis of behavior and health outcomes. J Health Care Poor Underserved. 2012;23(1):59-80. |
| 32 | Davies CA, Spence JC, Vandelanotte C, Caperchione CM, Mummery WK. Meta-analysis of internet-delivered interventions to increase physical activity levels. Int. 2012;9:52. |
| 33 | Denison E, Vist GE, Underland V, Berg RC. Interventions aimed at increasing the level of physical activity by including organised follow-up: a systematic review of effect. BMC Fam Pract. 2014;15:120. |
| 34 | Dishman RK, Buckworth J. Increasing physical activity: a quantitative synthesis. Med Sci Sports Exerc. 1996;28(6):706-19. |
| 35 | Dishman RK, Oldenburg B, O'Neal H, Shephard RJ. Worksite physical activity interventions. American Journal of Preventive Medicine. 1998;15(4):344-61. |
| 36 | Dunn AL. Getting started--a review of physical activity adoption studies. Br J Sports Med. 1996;30(3):193-9. |
| 37 | Eakin EG, Glasgow RE, Riley KM. Review of primary care-based physical activity intervention studies: effectiveness and implications for practice and future research. J. 2000;49(2):158-68. |
| 38 | Eakin EG, Lawler SP, Vandelanotte C, Owen N. Telephone interventions for physical activity and dietary behavior change: a systematic review. American Journal of Preventive Medicine. 2007;32(5):419-34. |
| 39 | Eaton CB, Menard LM. A systematic review of physical activity promotion in primary care office settings. Br J Sports Med. 1998;32(1):11-6. |
| 40 | Fanning J, Mullen SP, McAuley E. Increasing physical activity with mobile devices: a meta-analysis. J Med Internet Res. 2012;14(6):e161. |
| 41 | Fjeldsoe B, Neuhaus M, Winkler E, Eakin E. Systematic review of maintenance of behavior change following physical activity and dietary interventions. Health Psychol. 2011;30(1):99-109. |
| 42 | Foster C, Richards J, Thorogood M, Hillsdon M. Remote and web 2.0 interventions for promoting physical activity. Cochrane Database Syst Rev [Internet]. 2013; (9). Available from: http://onlinelibrary.wiley.com/doi/10.1002/14651858.CD010395.pub2/abstract. |
| 43 | Foster CE, Brennan G, Matthews A, McAdam C, Fitzsimons C, Mutrie N. Recruiting participants to walking intervention studies: a systematic review. Int. 2011;8:137. |
| 44 | Freak-Poli RL, Cumpston M, Peeters A, Clemes SA. Workplace pedometer interventions for increasing physical activity. Cochrane Database Syst Rev. 2013;4:CD009209. |
| 45 | George ES, Kolt GS, Duncan MJ, Caperchione CM, Mummery WK, Vandelanotte C, et al. A review of the effectiveness of physical activity interventions for adult males. Sports Med. 2012;42(4):281-300. |
| 46 | Ginis KA, Nigg CR, Smith AL. Peer-delivered physical activity interventions: an overlooked opportunity for physical activity promotion. Transl Behav Med. 2013;3(4):434-43. |
| 47 | Goode AD, Reeves MM, Eakin EG. Telephone-delivered interventions for physical activity and dietary behavior change: an updated systematic review. American Journal of Preventive Medicine. 2012;42(1):81-8. |
| 48 | Hanson S, Jones A. Is there evidence that walking groups have health benefits? A systematic review and meta-analysis. Br J Sports Med. 2015;49(11):1-7. |
| 49 | Hillsdon M, Thorogood M. A systematic review of physical activity promotion strategies. Br J Sports Med. 1996;30(2):84-9. |
| 50 | Hutchinson AD, Wilson C. Improving nutrition and physical activity in the workplace: a meta-analysis of intervention studies. Health Promot Internation. 2012;27(2):238-49. |
| 51 | Ickes MJ, Sharma M. A systematic review of physical activity interventions in Hispanic adults. J Environ Public Health. 2012;2012:156435. |
| 52 | Jackson NW, Howes FS, Gupta S, Doyle JL, Waters E. Interventions implemented through sporting organisations for increasing participation in sport. Cochrane Database Syst Rev. 2005(2):CD004812. |
| 53 | Jenkins A, Christensen H, Walker JG, Dear K. The effectiveness of distance interventions for increasing physical activity: a review. Am J Health Promot. 2009;24(2):102-17. |
| 54 | Kahn EB, Ramsey LT, Brownson RC, Heath GW, Howze EH, Powell KE, et al. The effectiveness of interventions to increase physical activity: A systematic review. American Journal of Preventive Medicine. 2002;22(4 SUPPL. 1):73-107. |
| 55 | Kang M, Marshall SJ, Barreira TV, Lee JO. Effect of pedometer-based physical activity interventions: a meta-analysis. Res Q Exerc Sport. 2009;80(3):648-55. |
| 56 | Kaushal N, Rhodes RE. The home physical environment and its relationship with physical activity and sedentary behavior: a systematic review. Preventive Medicine. 2014;67:221-37. |
| 57 | Keller C, Fleury J, Gregor-Holt N, Thompson T. Predictive ability of social cognitive theory in exercise research: an integrated literature review. Online J Knowl Synth Nurs. 1999;6:2. |
| 58 | Kroeze W, Werkman A, Brug J. A systematic review of randomized trials on the effectiveness of computer-tailored education on physical activity and dietary behaviors. Ann Behav Med. 2006;31(3):205-23. |
| 59 | Latimer AE, Brawley LR, Bassett RL. A systematic review of three approaches for constructing physical activity messages: What messages work and what improvements are needed? Int. 2010;7:36. |
| 60 | Lawlor DA, Hanratty B. The effect of physical activity advice given in routine primary care consultations: a systematic review. J Public Health Med. 2001;23(3):219-26. |
| 61 | Leavy JE, Bull FC, Rosenberg M, Bauman A. Physical activity mass media campaigns and their evaluation: a systematic review of the literature 2003-2010. Health Educ Res. 2011;26(6):1060-85. |
| 62 | Lemacks J, Wells BA, Ilich JZ, Ralston PA. Interventions for improving nutrition and physical activity behaviors in adult African American populations: a systematic review, January 2000 through December 2011. Prev Chronic Dis. 2013;10:E99. |
| 63 | Linke SE, Gallo LC, Norman GJ. Attrition and adherence rates of sustained vs. intermittent exercise interventions. Ann Behav Med. 2011;42(2):197-209. |
| 64 | Malik SH, Blake H, Suggs LS. A systematic review of workplace health promotion interventions for increasing physical activity. Br J Health Psychol. 2014;19(1):149-80. |
| 65 | Mama SK, McNeill LH, McCurdy SA, Evans AE, Diamond PM, Adamus-Leach HJ, et al. Psychosocial factors and theory in physical activity studies in minorities. Am J Health Behav. 2015;39(1):68-76. |
| 66 | Marcus BH, Ciccolo JT, Sciamanna CN. Using electronic/computer interventions to promote physical activity. Br J Sports Med. 2009;43(2):102-5. |
| 67 | Matson-Koffman DM, Brownstein JN, Neiner JA, Greaney ML. A site-specific literature review of policy and environmental interventions that promote physical activity and nutrition for cardiovascular health: what works? Am J Health Promot. 2005;19(3):167-93. |
| 68 | Mehta P, Sharma M. Internet and cell phone based physical activity interventions in adults. Archives of Exercise in Health & Disease. 2011;2(2):108-13. |
| 69 | Michie S, Abraham C, Whittington C, McAteer J, Gupta S. Effective techniques in healthy eating and physical activity interventions: a meta-regression. Health Psychol. 2009;28(6):690-701. |
| 70 | Mitchell MS, Goodman JM, Alter DA, John LK, Oh PI, Pakosh MT, et al. Financial incentives for exercise adherence in adults: systematic review and meta-analysis. American Journal of Preventive Medicine. 2013;45(5):658-67. |
| 71 | Morgan O. Approaches to increase physical activity: reviewing the evidence for exercise-referral schemes. Public Health. 2005;119(5):361-70. |
| 72 | Mozaffarian D, Afshin A, Benowitz NL, Bittner V, Daniels SR, Franch HA, et al. Population approaches to improve diet, physical activity, and smoking habits: a scientific statement from the American Heart Association. Circulation. 2012;126(12):1514-63. |
| 73 | Muller-Riemenschneider F, Reinhold T, Nocon M, Willich SN. Long-term effectiveness of interventions promoting physical activity: a systematic review. Preventive Medicine. 2008;47(4):354-68. |
| 74 | Murtagh EM, Nichols L, Mohammed MA, Holder R, Nevill AM, Murphy MH. The effect of walking on risk factors for cardiovascular disease: An updated systematic review and meta-analysis of randomised control trials. Preventive Medicine. 2015;72:34-43. |
| 75 | Neville LM, O'Hara B, Milat A. Computer-tailored physical activity behavior change interventions targeting adults: a systematic review. Int. 2009;6:30. |
| 76 | Nierkens V, Hartman MA, Nicolaou M, Vissenberg C, Beune EJ, Hosper K, et al. Effectiveness of cultural adaptations of interventions aimed at smoking cessation, diet, and/or physical activity in ethnic minorities. a systematic review. PLoS ONE. 2013;8(10):e73373. |
| 77 | Nocon M, Muller-Riemenschneider F, Nitzschke K, Willich SN. Review Article: Increasing physical activity with point-of-choice prompts--a systematic review. Scand J Public Health. 2010;38(6):633-8. |
| 78 | Norman GJ, Zabinski MF, Adams MA, Rosenberg DE, Yaroch AL, Atienza AA. A review of eHealth interventions for physical activity and dietary behavior change. American Journal of Preventive Medicine. 2007;33(4):336-45. |
| 79 | Ogilvie D, Egan M, Hamilton V, Petticrew M. Promoting walking and cycling as an alternative to using cars: systematic review. Bmj. 2004;329(7469):763. |
| 80 | Ogilvie D, Foster CE, Rothnie H, Cavill N, Hamilton V, Fitzsimons CF, et al. Interventions to promote walking: systematic review. Bmj. 2007;334(7605):1204. |
| 81 | Oja P, Titze S, Kokko S, Kujala UM, Heinonen A, Kelly P, et al. Health benefits of different sport disciplines for adults: systematic review of observational and intervention studies with meta-analysis. Br J Sports Med. 2015;49(7):434-40. |
| 82 | Orrow G, Kinmonth AL, Sanderson S, Sutton S. Effectiveness of physical activity promotion based in primary care: systematic review and meta-analysis of randomised controlled trials. Bmj. 2012;344:e1389. |
| 83 | Pavey TG, Taylor AH, Fox KR, Hillsdon M, Anokye N, Campbell JL, et al. Effect of exercise referral schemes in primary care on physical activity and improving health outcomes: systematic review and meta-analysis. Bmj. 2011;343:d6462. |
| 84 | Peng W, Crouse JC, Lin JH. Using active video games for physical activity promotion: a systematic review of the current state of research. Health Educ Behav. 2013;40(2):171-92. |
| 85 | Perez A, Fleury J, Keller C. Review of intervention studies promoting physical activity in Hispanic women. West J Nurs Res. 2010;32(3):341-62. |
| 86 | Priest N, Armstrong R, Doyle J, Waters E. Interventions implemented through sporting organisations for increasing participation in sport. Cochrane Database Syst Rev. 2008(3):CD004812. |
| 87 | Prince SA, Saunders TJ, Gresty K, Reid RD. A comparison of the effectiveness of physical activity and sedentary behaviour interventions in reducing sedentary time in adults: a systematic review and meta-analysis of controlled trials. Obes Rev. 2014;15(11):905-19. |
| 88 | Proper KI, Koning M, van der Beek AJ, Hildebrandt VH, Bosscher RJ, van Mechelen W. The effectiveness of worksite physical activity programs on physical activity, physical fitness, and health. Clin J Sport Med. 2003;13(2):106-17. |
| 89 | Rhodes RE, de Bruijn GJ. How big is the physical activity intention-behaviour gap? A meta-analysis using the action control framework. Br J Health Psychol. 2013;18(2):296-309. |
| 90 | Rhodes RE, Fiala B, Conner M. A review and meta-analysis of affective judgments and physical activity in adult populations. Ann Behav Med. 2009;38(3):180-204. |
| 91 | Rhodes RE, Pfaeffli LA. Mediators of physical activity behaviour change among adult non-clinical populations: a review update. Int. 2010;7:37. |
| 92 | Rhodes RE, Warburton DE, Murray H. Characteristics of physical activity guidelines and their effect on adherence: a review of randomized trials. Sports Med. 2009;39(5):355-75. |
| 93 | Richards J, Hillsdon M, Thorogood M, Foster C. Face-to-face interventions for promoting physical activity. Cochrane Database Syst Rev. 2013;9:CD010392. |
| 94 | Scheepers CE, Wendel-Vos GCW, den Broeder JM, van Kempen E, van Wesemael PJV, Schuit AJ. Shifting from car to active transport: A systematic review of the effectiveness of interventions. Transportation Research Part a-Policy and Practice. 2014;70:264-80. |
| 95 | Seron P, Lanas F, Pardo Hernandez H, Bonfill Cosp X. Exercise for people with high cardiovascular risk. Cochrane Database Syst Rev. 2014;8:CD009387. |
| 96 | Shilts MK, Horowitz M, Townsend MS. Goal setting as a strategy for dietary and physical activity behavior change: a review of the literature. Am J Health Promot. 2004;19(2):81-93. |
| 97 | Short CE, James EL, Plotnikoff RC, Girgis A. Efficacy of tailored-print interventions to promote physical activity: a systematic review of randomised trials. Int. 2011;8:113. |
| 98 | Simons-Morton DG, Calfas KJ, Oldenburg B, Burton NW. Effects of interventions in health care settings on physical activity or cardiorespiratory fitness. American Journal of Preventive Medicine. 1998;15(4):413-30. |
| 99 | Spana TM, Rodrigues RC, Lourenco LB, Mendez RD, Gallani MC. Integrative review: behavioral interventions for physical activity practice. Rev Lat Am Enfermagem. 2009;17(6):1057-64. |
| 100 | Stephens J, Allen J. Mobile phone interventions to increase physical activity and reduce weight: a systematic review. J Cardiovasc Nurs. 2013;28(4):320-9. |
| 101 | Sweet SN, Fortier MS. Improving physical activity and dietary behaviours with single or multiple health behaviour interventions? A synthesis of meta-analyses and reviews. Int J Environ Res Public Health. 2010;7(4):1720-43. |
| 102 | Taylor WC, Baranowski T, Young DR. Physical activity interventions in low-income, ethnic minority, and populations with disability. American Journal of Preventive Medicine. 1998;15(4):334-43. |
| 103 | Teufel-Shone NI, Fitzgerald C, Teufel-Shone L, Gamber M. Systematic review of physical activity interventions implemented with American Indian and Alaska Native populations in the United States and Canada. Am J Health Promot. 2009;23(6):S8-32. |
| 104 | van den Berg MH, Schoones JW, Vliet Vlieland TP. Internet-based physical activity interventions: a systematic review of the literature. J Med Internet Res. 2007;9(3):e26. |
| 105 | Vandelanotte C, Spathonis KM, Eakin EG, Owen N. Website-delivered physical activity interventions a review of the literature. American Journal of Preventive Medicine. 2007;33(1):54-64. |
| 106 | Wang CW, Chan CH, Ho RT, Chan JS, Ng SM, Chan CL. Managing stress and anxiety through qigong exercise in healthy adults: a systematic review and meta-analysis of randomized controlled trials. BMC Altern Med. 2014;14:8. |
| 107 | Waters L, Reeves M, Fjeldsoe B, Eakin E. Control Group Improvements in Physical Activity Intervention Trials and Possible Explanatory Factors: A Systematic Review. Journal of Physical Activity & Health. 2012;9(6):884-95. |
| 108 | Wendel-Vos W, Droomers M, Kremers S, Brug J, van Lenthe F. Potential environmental determinants of physical activity in adults: a systematic review. Obes Rev. 2007;8(5):425-40. |
| 109 | Westgarth C, Christley RM, Christian HE. How might we increase physical activity through dog walking?: A comprehensive review of dog walking correlates. Int. 2014;11:83. |
| 110 | Whitt-Glover MC, Keith NR, Ceaser TG, Virgil K, Ledford L, Hasson RE. A systematic review of physical activity interventions among African American adults: evidence from 2009 to 2013. Obes Rev. 2014;15 Suppl 4:125-45. |
| 111 | Whitt-Glover MC, Kumanyika SK. Systematic review of interventions to increase physical activity and physical fitness in African-Americans. Am J Health Promot. 2009;23(6):S33-56. |
| 112 | Williams G, Hamm MP, Shulhan J, Vandermeer B, Hartling L. Social media interventions for diet and exercise behaviours: a systematic review and meta-analysis of randomised controlled trials. BMJ Open. 2014;4(2):e003926. |
| 113 | Williams NH, Hendry M, France B, Lewis R, Wilkinson C. Effectiveness of exercise-referral schemes to promote physical activity in adults: systematic review. Br J Gen Pract. 2007;57(545):979-86. |
| 114 | Williams SL, French DP. What are the most effective intervention techniques for changing physical activity self-efficacy and physical activity behaviour--and are they the same? Health Educ Res. 2011;26(2):308-22. |
| 115 | Wong JY, Gilson ND, van Uffelen JG, Brown WJ. The effects of workplace physical activity interventions in men: a systematic review. Am j. 2012;6(4):303-13. |
| 116 | Yang L, Sahlqvist S, McMinn A, Griffin SJ, Ogilvie D. Interventions to promote cycling: systematic review. Bmj. 2010;341:c5293. |
|  |  |
|  |  |
|  | **Reason: Not a systematic review** |
| 1 | Balis LE, Strayer Iii T, Ramalingam NP, Wilson M, Harden SM. Open-access physical activity programs for older adults: A pragmatic and systematic review. Gerontologist. 2020;59(4):E268-E78. |
| 2 | Barton J, Pretty J. What is the best dose of nature and green exercise for improving mental health? A multi-study analysis. Environ Sci Technol. 2010;44(10):3947-55. |
| 3 | Bassuk SS, Manson JE. Physical activity and cardiovascular disease prevention in women: How much is good enough? Exercise and Sport Sciences Reviews. 2003;31(4):176-81. |
| 4 | Beitz R, Doren M. Physical activity and postmenopausal health. J Br Menopause Soc. 2004;10(2):70-4. |
| 5 | Boulton E, Hawley-Hague H, French DP, Mellone S, Zacchi A, Clemson L, et al. Implementing behaviour change theory and techniques to increase physical activity and prevent functional decline among adults aged 61–70: The PreventIT project. Prog Cardiovasc Dis. 2019;62(2):147-56. |
| 6 | Brand T, Pischke CR, Steenbock B, Schoenbach J, Poettgen S, Samkange-Zeeb F, et al. What works in community-based interventions promoting physical activity and healthy eating? A review of reviews. Int J Environ Res Public Health. 2014;11(6):5866-88. |
| 7 | Collado-Mateo D, Lavín-Pérez AM, Peñacoba C, Del Coso J, Leyton-Román M, Luque-Casado A, et al. Key Factors Associated with Adherence to Physical Exercise in Patients with Chronic Diseases and Older Adults: An Umbrella Review. International journal of environmental research and public health. 2021;18(4). |
| 8 | Conn VS, Isaramalai S, Banks-Wallace JA, Ulbrich S, Cochran J. Evidence-based interventions to increase physical activity among older adults. Activities, Adaptation & Aging. 2002;27(2):39-52. |
| 9 | Di Lorito C, Long A, Byrne A, Harwood RH, Gladman JRF, Schneider S, et al. Exercise interventions for older adults: A systematic review of meta-analyses. Journal of sport and health science. 2020. |
| 10 | Hind D, Scott EJ, Copeland R, Breckon JD, Crank H, Walters SJ, et al. A randomised controlled trial and cost-effectiveness evaluation of "booster" interventions to sustain increases in physical activity in middle-aged adults in deprived urban neighbourhoods. BMC Public Health. 2010;10:3. |
| 11 | Kenny GP, Yardley JE, Martineau L, Jay O. Physical work capacity in older adults: implications for the aging worker. Am J Ind Med. 2008;51(8):610-25. |
| 12 | Lim SER, Cox NJ, Roberts HC. THE EFFECTIVENESS OF VOLUNTEER-LED PHYSICAL ACTIVITY INTERVENTIONS IN IMPROVING HEALTH OUTCOMES FOR COMMUNITY-DWELLING OLDER PEOPLE: A SYSTEMATIC REVIEW...British Geriatrics Society Autumn Meeting, November 6-8, 2019, Leicester, England. Age & Ageing. 2020;49:30-. |
| 13 | Morelhao PK, Oliveira CB, Franco MR. Interventions to increase physical activity among older adults (PEDro synthesis). Br J Sports Med. 2017;51(18):1375-6. |
| 14 | Otmanowski JA, Chase JAD. Systematic Review and Meta-Analysis of Primary Care-Based Physical Activity Interventions Among Older Adults. Journal of Aging and Physical Activity. 2022;30(5):842-56. |
| 15 | Williams AD. Use of a text messaging program to promote adherence to daily physical activity guidelines: A review of the literature. Bariatric Nursing and Surgical Patient Care. 2012;7(1):13-6. |
|  |  |
|  |  |
|  | **Reason: Wrong outcome** |
| 1 | Astrup A. Physical activity and weight gain and fat distribution changes with menopause: current evidence and research issues. Med Sci Sports Exerc. 1999;31(11 Suppl):S564-7. |
| 2 | Balis LE, Strayer T, 3rd, Ramalingam N, Wilson M, Harden SM. Open-Access Physical Activity Programs for Older Adults: A Pragmatic and Systematic Review. Gerontologist. 2018;10:10. |
| 3 | Bergevi J, Andermo S, Woldamanuel Y, Johansson U-B, Hagstromer M, Rossen J. User Perceptions of eHealth and mHealth Services Promoting Physical Activity and Healthy Diets: Systematic Review. JMIR human factors. 2022;9(2):e34278. |
| 4 | Bong May Ing J, Singh DKA, Tan MP, Adam Bujang M, Tiong IK, Whitney J, et al. Group-based exercise interventions for community-dwelling older people in Southeast Asia: A systematic review. Australasian journal on ageing. 2023. |
| 5 | Burton E, Farrier K, Hill KD, Codde J, Airey P, Hill AM. Effectiveness of peers in delivering programs or motivating older people to increase their participation in physical activity: Systematic review and meta-analysis. Journal of Sports Sciences. 2018;36(6):666-78. |
| 6 | Chaabene H, Prieske O, Herz M, Moran J, Höhne J, Kliegl R, et al. Home-based exercise programmes improve physical fitness of healthy older adults: A PRISMA-compliant systematic review and meta-analysis with relevance for COVID-19. Ageing research reviews. 2021;67:101265. |
| 7 | Chase JAD, Otmanowski J, Rowland S, Cooper PS. A systematic review and meta-analysis of interventions to reduce sedentary behavior among older adults. Transl Behav Med. 2020;10(5):1078-85. |
| 8 | Conn VS, Hafdahl A, Phillips LJ, Ruppar TM, Chase JA. Impact of physical activity interventions on anthropometric outcomes: systematic review and meta-analysis. J Prim Prev. 2014;35(4):203-15. |
| 9 | Cruz-Ferreira A, Fernandes J, Laranjo L, Bernardo LM, Silva A. A systematic review of the effects of pilates method of exercise in healthy people. Arch Phys Med Rehabil. 2011;92(12):2071-81. |
| 10 | Ismail NA, Hashim HA, Ahmad Yusof H. Physical Activity and Exergames Among Older Adults: A Scoping Review. Games for health journal. 2022;11(1):1-17. |
| 11 | Lee MS, Ernst E. Systematic reviews of t'ai chi: an overview. Br J Sports Med. 2012;46(10):713-8. |
| 12 | Ng YL, Hill KD, Levinger RP, Burton E. Effectiveness of Outdoor Exercise Parks on Health Outcomes in Older Adults-A Mixed-Methods Systematic Review and Meta-Analysis. Journal of aging and physical activity. 2020:1-13. |
| 13 | Paterson DH, Warburton DE. Physical activity and functional limitations in older adults: a systematic review related to Canada's Physical Activity Guidelines. Int. 2010;7:38. |
| 14 | Sherrington C, Fairhall N, Kwok W, Wallbank G, Tiedemann A, Michaleff ZA, et al. Evidence on physical activity and falls prevention for people aged 65+ years: systematic review to inform the WHO guidelines on physical activity and sedentary behaviour. The international journal of behavioral nutrition and physical activity. 2020;17(1):144. |
| 15 | Solis-Navarro L, Gismero A, Fernández-Jané C, Torres-Castro R, Solá-Madurell M, Bergé C, et al. Effectiveness of home-based exercise delivered by digital health in older adults: a systematic review and meta-analysis. Age and ageing. 2022;51(11). |
| 16 | Valdes-Badilla P, Gutierrez-Garcia C, Perez-Gutierrez M, Vargas-Vitoria R, Lopez-Fuenzalida A. Effects of Physical Activity Governmental Programs on Health Status in Independent Older Adults: A Systematic Review. J Aging Phys Activity. 2018:1-32. |
| 17 | Wanner M, Gotschi T, Martin-Diener E, Kahlmeier S, Martin BW. Active transport, physical activity, and body weight in adults: a systematic review. American Journal of Preventive Medicine. 2012;42(5):493-502. |
| 18 | Weber Buchholz S, Wilbur J, Halloway S, McDevitt JH, Schoeny ME. Physical activity intervention studies and their relationship to body composition in healthy women. Annu Rev Nurs Res. 2013;31:71-142. |
|  |  |
|  |  |
|  | **Reason: Wrong population** |
| 1 | Anthony K, Connel L, Logan P, Gladman J, Masud T. Chair based exercise in frail older people: A systematic review. Journal of Aging and Physical Activity. 2012;20:S219-S20. |
| 2 | Ashworth NL, Chad KE, Harrison EL, Reeder BA, Marshall SC. Home versus center based physical activity programs in older adults. Cochrane Database Syst Rev. 2005(1):CD004017. |
| 3 | Baranowski T, Lyons EJ. Scoping Review of Pokemon Go: Comprehensive Assessment of Augmented Reality for Physical Activity Change. Games for health journal. 2020;9(2):71-84. |
| 4 | Chase JA. Systematic review of physical activity intervention studies after cardiac rehabilitation. J Cardiovasc Nurs. 2011;26(5):351-8. |
| 5 | Chaudhry UAR, Wahlich C, Fortescue R, Cook DG, Knightly R, Harris T. The effects of step-count monitoring interventions on physical activity: Systematic review and meta-analysis of community-based randomised controlled trials in adults. Int J Behav Nutr Phys Act. 2020;17(1). |
| 6 | Chen C, Finne E, Kopp A, Jekauc D. What Intervention Techniques Are Effective in Changing Positive Affective Variables and Physical Activity? A Systematic Review and Meta-Analysis. Front Psychol. 2021;12. |
| 7 | Chu AH, Koh D, Moy FM, Muller-Riemenschneider F. Do workplace physical activity interventions improve mental health outcomes? Occup Med (Oxf). 2014;64(4):235-45. |
| 8 | Clark IN, Taylor NF, Baker F. Music interventions and physical activity in older adults: a systematic literature review and meta-analysis. J Rehabil Med. 2012;44(9):710-9. |
| 9 | Clark IN, Taylor NF, Peiris CL. Music listening interventions for physical activity: a systematic review and meta-analysis of randomised controlled trials. Disability and rehabilitation. 2022:1-8. |
| 10 | D'Amore C, Reid JC, Chan M, Fan S, Huang A, Louie J, et al. Interventions Including Smart Technology Compared With Face-to-face Physical Activity Interventions in Older Adults: Systematic Review and Meta-analysis. J Med Internet Res. 2022;24(10). |
| 11 | Daniel M, Buchholz S, Fogg L. Physical Activity in South Asian Indians: A Systematic Review of Randomized Controlled Trials. West J Nurs Res. 2023;45(4):363-74. |
| 12 | Geohagen O, Hamer L, Lowton A, Guerra S, Milton-Cole R, Ellery P, et al. The effectiveness of rehabilitation interventions including outdoor mobility on older adults' physical activity, endurance, outdoor mobility and falls-related self-efficacy: systematic review and meta-analysis. Age and ageing. 2022;51(6). |
| 13 | Guerra PH, Sposito LAC, da Costa FF, Fermino RC, Papini CB, Rech CR. Effectiveness of the 5A Counseling Model-Based Interventions on Physical Activity Indicators in Adults: A Systematic Review. Behavioral sciences (Basel, Switzerland). 2023;13(6). |
| 14 | Katigbak C, Flaherty E, Chao Y-Y, Nguyen T, Cheung D, Yiu-Cho Kwan R. A Systematic Review of Culturally Specific Interventions to Increase Physical Activity for Older Asian Americans. The Journal of cardiovascular nursing. 2018;33(4):313-21. |
| 15 | Lim S, Lee WK, Tan A, Chen M, Tay CT, Sood S, et al. Peer-supported lifestyle interventions on body weight, energy intake, and physical activity in adults: A systematic review and meta-analysis. Obesity reviews : an official journal of the International Association for the Study of Obesity. 2021;22(12):e13328. |
| 16 | Loya JC. Systematic Review of Physical Activity Interventions and Hispanic Adults. Hispanic health care international : the official journal of the National Association of Hispanic Nurses. 2018;16(4):1540415318809427. |
| 17 | Luong M-LN, Hall M, Bennell KL, Kasza J, Harris A, Hinman RS. The Impact of Financial Incentives on Physical Activity: A Systematic Review and Meta-Analysis. American journal of health promotion : AJHP. 2021;35(2):236-49. |
| 18 | Lynch C, Bird S, Lythgo N, Selva-Raj I. Changing the Physical Activity Behavior of Adults With Fitness Trackers: A Systematic Review and Meta-Analysis. Am J Health Promot. 2020;34(4):418-30. |
| 19 | Madigan CD, Fong M, Howick J, Kettle V, Rouse P, Hamilton L, et al. Effectiveness of interventions to maintain physical activity behavior (device-measured): Systematic review and meta-analysis of randomized controlled trials. Obesity reviews : an official journal of the International Association for the Study of Obesity. 2021;22(10):e13304. |
| 20 | McGrane N, Galvin R, Cusack T, Stokes E. Addition of motivational interventions to exercise and traditional physiotherapy: a review and meta-analysis. Physiotherapy. 2015;101(1):1-12. |
| 21 | Miller KJ, Adair BS, Pearce AJ, Said CM, Ozanne E, Morris MM. Effectiveness and feasibility of virtual reality and gaming system use at home by older adults for enabling physical activity to improve health-related domains: a systematic review. Age & Ageing. 2014;43(2):188-95. |
| 22 | Pradal-Cano L, Lozano-Ruiz C, Pereyra-Rodriguez JJ, Saigi-Rubio F, Bach-Faig A, Esquius L, et al. Using Mobile Applications to Increase Physical Activity: A Systematic Review. International journal of environmental research and public health. 2020;17(21). |
| 23 | Richards J, Thorogood M, Hillsdon M, Foster C. Face-to-face versus remote and web 2.0 interventions for promoting physical activity. Cochrane Database Syst Rev. 2013;9:CD010393. |
| 24 | Ryde GC, Gilson ND, Burton NW, Brown WJ. Recruitment rates in workplace physical activity interventions: characteristics for success. Am J Health Promot. 2013;27(5):e101-12. |
| 25 | To QG, Chen TT, Magnussen CG, To KG. Workplace physical activity interventions: a systematic review. Am J Health Promot. 2013;27(6):e113-23. |
| 26 | Vetrovsky T, Borowiec A, Jurík R, Wahlich C, Smigielski W, Steffl M, et al. Do physical activity interventions combining self-monitoring with other components provide an additional benefit compared with self-monitoring alone? A systematic review and meta-analysis. Br J Sports Med. 2022;56(23):1366-74. |
| 27 | Wahlich C, Chaudhry UAR, Fortescue R, Cook DG, Hirani S, Knightly R, et al. Effectiveness of adult community-based physical activity interventions with objective physical activity measurements and long-term follow-up: A systematic review and meta-analysis. BMJ Open. 2020;10(5). |
| 28 | Yang PY, Ho KH, Chen HC, Chien MY. Exercise training improves sleep quality in middle-aged and older adults with sleep problems: a systematic review. J Physiother. 2012;58(3):157-63. |
