# Supplementary material for: Physical activity interventions for older adults – an overview of systematic reviews
Source: BMC Public Health. 2026 Jan 6;26:205. doi: 10.1186/s12889-025-25002-2 (PMC12805782; doi:10.1186/s12889-025-25002-2)
Supplement: Supplementary file 5 — Supplementary Material 5. PROGRESS-plus [file 12889_2025_25002_MOESM5_ESM.pdf]

Review	Progress															Plus								
	Place of residence							Ethnic background	Professional, skilled, unskilled, unempl	Male or female	Religious background	Years in and/or level of education attained	Neighbourhood / community / family	Income-related measure e.g. means-tested	SES and other income-related measures	Age range	Disability		Sexual orientation	Other vulnerable and socially				Total Score
	Setting				Rural/urban	Country/state	Area deprivation										Existence of physical or / sensory	Emotional / mental disability		Elderly / infirm	Dementia	Widows / single occupancy	Social isolated	
	Living in the community	Sheltered housing	Housing with care	Care home																				
Meta analyses																								
Chase (2015)								✔ ♟		✔ ♟						✔ ♟								3
Conn *et al.* (2002)	✔ ♟			✔ ♟				✔ ♟		✔ ♟						✔ ♟				✔ ♟				6
Cooper *et al.* (2018)	✔ ♟																							1
French *et al.* (2014)	✔ ♟			✔ ♟				✔ ♟		✔ ♟						✔ ♟								5
Grande et al. (2020)	✔ ♟					✔ ♟										✔ ♟								3
Hobbs et al. (2013) & O'Brien et al. (2015)	✔ ♟					✔ ♟				✔ ♟						✔ ♟	✔ ♟							5
Kassavou *et al.* (2013)	✔ ♟			✔ ♟	✔ ♟					✔ ♟						✔ ♟								5
Kwan et al. (2020)																✔ ♟								1
Larsen et al. (2019)						✔ ♟				✔ ♟						✔ ♟								3
Liu et al. (2020)	✔ ♟					✔ ♟				✔ ♟						✔ ♟								4
Merom et al. (2021)	✔ ♟					✔ ♟	✔ ♟		✔ ♟	✔ ♟					✔ ♟	✔ ♟								7
Nunez de Arenas-Arroyo et al. (2021)						✔ ♟				✔ ♟						✔ ♟								3
Oliveira *et al.* (2017)	✔ ♟				✔ ♟					✔ ♟						✔ ♟				✔ ♟				5
Oliveira et al. (2020)	✔ ♟					✔ ♟				✔ ♟						✔ ♟								4
Sansano-Nadal et al. (2019)	✔ ♟							✔ ♟		✔ ♟						✔ ♟								4
Stockwell et al. (2019)	✔ ♟					✔ ♟				✔ ♟						✔ ♟								4
Wu et al. (2023)						✔ ♟				✔ ♟						✔ ♟								3
Yerrakalva et al. (2019)	✔ ♟					✔ ♟				✔ ♟						✔ ♟								4
TOTAL	16	0	0	3	2	12	1	4	1	17	0	0	0	0	1	20	1	0	0	2	0	0	0	
Narrative systematic reviews																								
Baxter *et al.* (2016)						✔ ♟		✔ ♟		✔ ♟		✔ ♟			✔ ♟	✔ ♟								6
Conn *et al.* (2003)	✔ ♟			✔ ♟				✔ ♟		✔ ♟						✔ ♟				✔ ♟				6
Cyarto *et al.* (2004)	✔ ♟			✔ ♟						✔ ♟			✔ ♟			✔ ♟								5
Elavsky et al. (2019)										✔ ♟						✔ ♟								2
Goethals et al. (2020)						✔ ♟																		1
Jansen *et al.* (2015)				✔ ♟												✔ ♟	✔ ♟	✔ ♟		✔ ♟	✔ ♟			6
Katigbak et al. (2018)	✔ ♟					✔ ♟		✔ ♟																3
Knight et al. (2022)	✔ ♟					✔ ♟				✔ ♟						✔ ♟								4
Lim et al. (2021) *volunteer-led*	✔ ♟		✔ ♟	✔ ♟		✔ ♟										✔ ♟								5
Moore *et al.* (2016)	✔ ♟			✔ ♟	✔ ♟			✔ ♟								✔ ♟	✔ ♟							6
Muellmann *et al.* (2018)						✔ ♟		✔ ♟		✔ ♟		✔ ♟			✔ ♟	✔ ♟								6
Muller and Khoo (2014)	✔ ♟					✔ ♟				✔ ♟		✔ ♟			✔ ♟	✔ ♟								6
Neidrick *et al.* (2012)						✔ ♟																		1
Ostrander *et al.* (2014)								✔ ♟								✔ ♟								2
Pedersen et al. (2022)	✔ ♟				✔ ♟	✔ ♟		✔ ♟		✔ ♟						✔ ♟								6
Stevens *et al.* (2014)	✔ ♟				✔ ♟	✔ ♟				✔ ♟						✔ ♟								5
Van Der kangsdpn *et al.* (2002)	✔ ♟			✔ ♟		✔ ♟		✔ ♟		✔ ♟		✔ ♟		✔ ♟		✔ ♟								8
**TOTAL**	10	0	1	6	3	11	0	8	0	10	0	4	1	1	3	14	2	1	0	2	1	0	0	
