# Supplementary figures and images for: Physical activity interventions for older adults – an overview of systematic reviews
Source: BMC Public Health. 2026 Jan 6;26:205. doi: 10.1186/s12889-025-25002-2 (PMC12805782; doi:10.1186/s12889-025-25002-2)

Country of includes


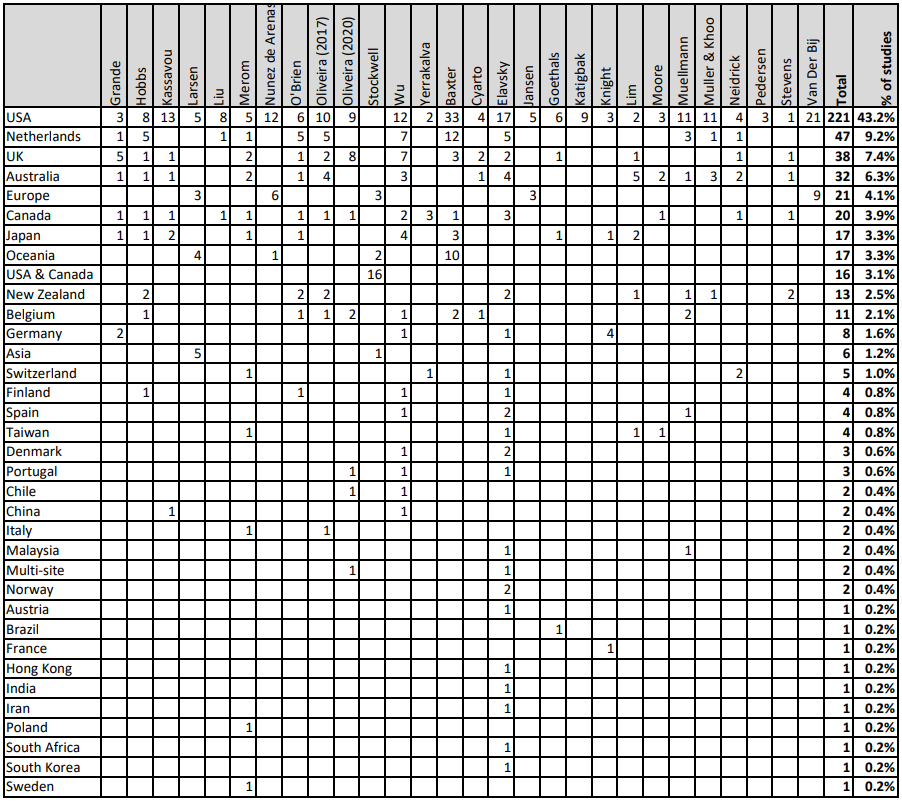


Continent of includes


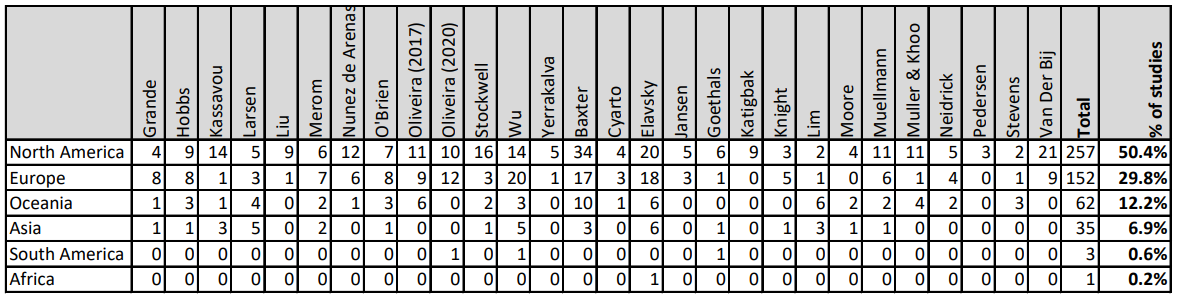

Supplement: Supplementary file 7 — Supplementary Material 7. Country / continent of included studies [file 12889_2025_25002_MOESM7_ESM.docx]
